# Exploring serum biomarkers in paediatric psychiatry: the impact of NfL and GFAP

**DOI:** 10.1038/s41398-026-04347-1

**Published:** 2026-08-26

**Authors:** Marc Pawlitzki, Lars Masanneck, Laura Schlarbaum, Nicole Jankovic, Stefan Bittner, Falk Steffen, Jens Kuhle, Pascal Benkert, Jonathan Repple, Judith Bühlmeier, Anke Hinney, Johannes Hebebrand, Raphael Hirtz, Sven G. Meuth, Lars Libuda, Manuel Föcker

**Affiliations:** 1 Department of Neurology, Medical Faculty University Hospital Düsseldorf, Düsseldorf, Germany; 2 Paderborn University, Faculty of Natural Sciences, Institute of Nutrition, Consumption and Health, Paderborn, Germany; 3 Institute for Integrative Health Care and Health Promotion, Witten/Herdecke University, Alfred-Herrhausen-Str. 44, 58455 Witten, Germany; 4 Department of Neurology, Focus Program Translational Neuroscience (FTN) and Immunotherapy (FZI), Rhine Main Neuroscience Network (RMN2), Medical Center, Johannes Gutenberg University Mainz, Mainz, Germany; 5 Multiple Sclerosis Centre and Research Center for Clinical Neuroimmunology and Neuroscience (RC2NB), Departments of Biomedicine and Clinical Research, University Hospital and University of Basel, Basel, Switzerland; 6 Goethe University Frankfurt, University Hospital, Department of Psychiatry, Psychosomatic Medicine and Psychotherapy, Frankfurt, Germany; 7 Institute for Translational Psychiatry, University of Münster, Münster, Germany; 8 Department of Child and Adolescent Psychiatry, Psychosomatics and Psychotherapy, University Hospital Essen, University of Duisburg-Essen, Essen, Germany; 9 Center for Translational Neuro- and Behavioral Sciences, University Hospital Essen, Essen, Germany; 10 Section of Molecular Genetics in Mental Disorders, University Hospital Essen, Essen, Germany; 11 Institute of Sex and Gender-Sensitive Medicine, University Hospital Essen, Essen, Germany; 12 Helios University Medical Centre Wuppertal - Children’s Hospital, Witten/Herdecke University, Wuppertal, Germany; 13 Department of Child and Adolescent Psychiatry, University Hospital Münster, Münster, Germany; 14 LWL University Hospital Hamm for Child and Adolescent Psychiatry, Psychotherapy and Psychosomatics, Ruhr University-Bochum, Campus Gütersloh, Gütersloh, Germany

**Keywords:** Diagnostic markers, Predictive markers

## Abstract

Serum neurofilament light chain (sNfL) and glial fibrillary acidic protein (GFAP) are biomarkers of neuroaxonal and astrocytic damage but remain understudied in adolescent psychiatric populations. This study investigated sNfL and GFAP levels in 412 adolescents diagnosed with anorexia nervosa (AN) (*n* = 52), depression (*n* = 237), and other psychiatric disorders (*n* = 123). We assessed their diagnostic utility, correlation with disease severity, and longitudinal changes during AN treatment. Biomarkers were measured using Single Molecule Array technology, with Z-scores derived from reference datasets. Compared to population norms, both biomarkers were elevated in AN (sNfL: 1.15 ± 1.17; GFAP: 1.50 ± 0.84) and in depression (sNfL: 0.34 ± 1.10; GFAP: 0.58 ± 1.00). Patients with AN showed significantly higher biomarker levels than those with depression or other psychiatric disorders; importantly, this distinction remained evident in sensitivity analyses restricted to underweight individuals with depression. In AN, sNfL levels correlated with baseline weight loss (β = −0.45, R² = 0.20) and declined significantly during treatment, while GFAP changes were less pronounced. Neither marker correlated with depressive symptom severity. Bootstrapped ROC analyses showed moderate-to-good discriminatory power (AUCs 0.70–0.84) for distinguishing AN from depression. These findings suggest that neuroaxonal and astrocytic stress is a component of adolescent psychopathology, particularly in AN. sNfL appears sensitive to starvation-related neurobiological changes, with levels normalizing alongside weight restoration. GFAP showed similar but less robust trends. Accordingly, the observed biomarker changes reflect more than underweight alone, supporting a potential role in differential diagnosis and treatment monitoring.

## Background

Adolescence is a critical period for psychiatric disorders such as depression and eating disorders, often undiagnosed due to stigma, parental misrecognition, limited screening, and restricted healthcare access [1]. Symptom overlap hinders distinguishing psychiatric disorders, with depressive symptoms common in eating disorders [2] and vice versa loss of appetite potentially causing weight loss in depression. Thus, psychiatric disorders are highly diverse and often difficult to delineate clearly. Depression, for example, includes various subtypes with distinct biological mechanisms, complicating the search for uniform biomarkers [3].

Notably, psychiatric disorders are closely associated with compromised cognitive functions [4], often linked to changes in brain structure [5]. MRI studies in anorexia nervosa (AN) show reduced grey matter volume in underweight patients, likely due to starvation, with some evidence of persistent changes post-recovery [6, 7]. These findings highlight the need for early, holistic treatment addressing both physical and mental health.

Neurofilament light chain (NfL), a neuronal cytoskeletal protein detectable in blood and cerebrospinal fluid, serves as an indicator of axonal damage caused by neuroinflammation or injury [8, 9]. Serum NfL (sNfL) shows promise as a biomarker for monitoring neuronal damage in neurological diseases [10]. In contrast, recent studies report inconsistent findings on sNfL elevation in adults with depression but suggest its association with cognitive decline [11, 12]. Notably, sNfL levels are significantly elevated in acute AN compared to healthy controls and individuals who had recovered from AN [13].

Glial Fibrillary Acidic Protein (GFAP) is a potential biomarker for astrocytic activation and brain injury [14]. GFAP has rarely been studied in psychiatric conditions, with conflicting results [11, 15]. *Steinacker et al*. (2021) suggest serum GFAP may distinguish adults with major depression from healthy controls and indicate depression severity [14].

Both biomarkers offer complementary insights into brain pathology, providing a convenient alternative to MRI. Combined with clinical assessments, sNfL and GFAP may help track disease progression and treatment response, though most studies have focused on adults [16]. Beyond their biological characterization, an important unresolved question is whether sNfL and GFAP capture clinically meaningful neurobiological dimensions across adolescent psychiatric disorders. In this context, cross-diagnostic comparisons are useful not because these potential biomarkers are expected to replace clinically established diagnoses, but because they may help determine whether AN is associated with a biologically interpretable biomarker signal consistent with neuroaxonal and astrocytic stress relative to closely related psychiatric comparators. We therefore compared biomarker levels across adolescent psychiatric disorders, with a focus on AN and depression, and examined associations with clinical severity and longitudinal change during AN treatment.

## Methods

Detailed methods, including therapy, diagnostics, assessments, and statistics, are in Supplementary Methods.

### Study sample

Our data compromised three clinical cohorts of patients (aged 11–18 years) recruited between 2013 and 2020 from the Department of Child and Adolescent Psychiatry, Psychosomatics and Psychotherapy (LVR-Klinikum Essen) at the University Hospital Essen, Germany. Overall, we enrolled *N* = 429 patients with clinically confirmed psychiatric diagnoses (ICD-10, Supplementary Methods), of whom 69 had AN (F50) and 237 had depression (F32). Among individuals with AN, 17 patients with comorbid depression - confirmed through clinical diagnosis or the Beck Depression Inventory II (BDI-II sum score > 13) - were excluded to focus on disease-specific changes and facilitate comparison with patients diagnosed solely with depression, increase disorder specificity and facilitate clearer between-group comparisons, acknowledging that this approach may reduce ecological validity given the high rates of comorbidity in clinical practice.

The group with “other psychiatric disorders” (*N* = 123) comprised a heterogeneous set of diagnoses and was included as a pragmatic comparator rather than for disorder-specific analyses. This group consisted of 55 patients with anxiety disorders or phobias (F40-43), 22 with cannabis dependence (F12), 16 with hyperactivity disorder (F90), and 30 with disorders of social behaviour (F91), all without comorbid AN or depression (Supplementary Fig. 1). Accordingly, the final sample included 412 patients.

Moreover, patients were clinically assessed as part of routine care, and no relevant neurological or severe somatic disorders known to affect sNfL or GFAP, such as epilepsy, multiple sclerosis, or chronic inflammatory disorders including rheumatologic or inflammatory bowel diseases, were documented in the study cohort.

Longitudinal data were available for a subset of AN patients from the first study, all receiving standard multimodal treatment. Based on prior work [17], we analyzed biomarker levels in the AN subgroup at three stages: acute starvation at admission (T0, *N* = 52), halfway to target weight (T1, *N* = 26), and near target weight per German guidelines (T2, *N* = 22), with complete data available for *N* = 17 [17, 18].

### sNfL and GFAP measures

The Single Molecule Array (SIMOA) HD-1 analyzer (Quanterix®) was utilized to determine NfL and GFAP levels in serum using the Neurology 2-Plex B Kit (Quanterix®) according to the manufacturer’s instructions. The sNfL and GFAP values were measured with one lot (503227). SNfL Z scores were calculated as described before [19]. As the current sNfL pediatric reference database [9] does not account for BMI variation, which we consider to be substantial in our psychiatric cohort, we decided to use the adult reference database [19] instead. In general, the adult database allows the calculation of age- and BMI-adjusted sNfL Z-scores, but does not further discriminate the effects of age on sNfL for individuals under 17 years of age. Given the relatively small age range in our sample (11–19 years) and the comparably small effect of age in that range [20], we estimate the potential bias to be likely modest compared with missing BMI adjustment and conducted a set of sensitivity analyses (see below).

For GFAP, a reference database considering sex, age, and body weight was recently published [21]. For participants < 17 years, we applied the reference values for 17-year-olds (youngest age in reference database) to calculate the experimental Z-scores, given the limited understanding of dynamics in adolescents [20].

Analyses using raw sNfL and GFAP values were primarily performed without correcting for covariates such as age, sex and BMI in contrast to Z-score results. A sensitivity analysis with such internal corrections is described further below.

### Statistical analysis

Statistical analyses were conducted in Python 3.8.15 using SciPy (v1.9.3) and NumPy (v1.23.5) for calculations, and matplotlib (v3.6.2) with seaborn (v0.12.0) for visualizations. Normality and variance were assessed via Shapiro-Wilk and Levene’s tests, guiding parametric (ANOVA, t-tests) or non-parametric (Kruskal-Wallis, Dunn’s tests) approaches. Categorical variables were compared using Chi-Square tests with Bonferroni correction. Biomarker Z-scores were tested against reference populations via one-sample t-tests. Tukey’s HSD followed ANOVA, and Dunn’s test with Bonferroni correction followed Kruskal-Wallis. Only significant post-hoc results are shown in visualizations.

To assess whether biomarker differences were driven by weight despite using BMI-adjusted Z-scores, we analyzed BMI-SDS correlations across diagnoses using linear regression, correcting *p*-values via Bonferroni. For AN severity, regression models examined BMI-SDS change (premorbid to baseline) as a predictor of biomarker Z-scores. For depression severity, biomarker levels were tested against BDI-II scores. To analyze longitudinal sNfL and GFAP trajectories in AN, Repeated Measures ANOVA (parametric data) or Friedman tests (non-parametric data) were applied to Z-scores and raw values, considering only patients with all three timepoints (T0, T1, T2). Post-hoc comparisons followed standard methods. Additionally, a linear mixed-effects model (“Serum Biomarker ~ elapsed months since admission + Clinical Timepoint + (1|Patient_ID)”) included all patients, accounting for random intercepts. Elapsed time was calculated from the exact sampling dates as months since admission. For descriptive readability, follow-up timing at T1 and T2 is reported in days after admission. “Patient_ID” was included to account for individual intercepts, ensuring all longitudinal data were utilized while modeling within-subject variability and minimizing data loss. To distinguish AN from underweight depression, 1000-fold bootstrapped ROC analyses assessed sNfL and GFAP, calculating AUC, sensitivity, and specificity with confidence intervals from bootstrap distributions.

### Further sensitivity analyses

We performed several additional sensitivity analyses. First, within the aggregated subgroup of patients with other psychiatric disorders, we compared internalizing presentations (anxiety/phobia) with externalizing presentations (attention-deficit/hyperactivity disorder and disorder of social behavior) and additionally explored the exact primary subgroups. Second, we repeated the main analyses using raw sNfL and GFAP values after log10 transformation and internal normalization with age and BMI-SDS compared to an internal control subset of 25 healthy adolescent females (as the AN patients) [22]. This served as the primary normalization approach, with a full-baseline sex-adjusted model used as a technical sensitivity check.

As a further sensitivity analysis in the depression cohort, we assessed potential confounding by medication exposure using binary indicators for antidepressant use and psychopharmacological medication (included antidepressants) use (yes/no). These variables were tested for imbalance across depression weight groups and were entered separately into linear models of baseline sNfL and GFAP Z-scores adjusted for weight group, age, and sex.

The adjusted *p*-values are reported for the post-hoc and all other tests. Throughout this manuscript the following asterixis annotation is used for *p*-values: *p* ≤ 0.05 (*), *p* ≤ 0.01 (**), *p* ≤ 0.001 (***), *p* ≤ 0.0001 (****).

Full methodological details, including threshold calculations and statistical thresholds, are provided in the Supplementary Methods.

## Results

Table 1 displays the demographic and clinical characteristics of the three groups, revealing notable differences in age, sex and BMI.

**Table 1:** Median (inter-quartile range (IQR)) age at baseline was 15.4 (14.6–16.4) years for the AN group, 16.1 (14.8–17.1) for the depression group and 15.5 (13.3–16.8) for patients with other psychiatric disorders.

	Anorexia		Depression		Other Psychiatric Disorders		
	n		n		n		
Number (n)	52		237		123		p
Age [years] (median, IQR)	52/52	15.4 (14.6–16.4)	237/237	16.1 (14.8–17.1)	123/123	15.5 (13.3–16.8)	0.0015
Sex [number of females] (n, %)	52/52	51 (98.1)	237/237	186 (78.6)	123/123	51 (41.5)	<0.0001
Baseline BMI [kg/m²] (median, IQR)	52/52	15.5 (14.6–16.2)	236/237	22.3 (19.7–26.5)	123/123	21.2 (17.8–25.0)	<0.0001
Baseline BMI SDS [z-score] (mean, std)	52/52	−2.90 (1.2)	236/237	0.22 (1.45)	123/123	0.06 (1.52)	<0.0001
Baseline sNfL [pg/ml] (median, IQR)	52/52	9.8 (6.8–12.5)	237/237	5.1 (3.9–6.4)	123/123	4.6 (3.9–5.9)	<0.0001
Adjusted baseline sNfL [z-score] (mean, std)	52/52	1.15 (1.17)	237/237	0.35 (1.10)	123/123	0.01 (1.18)	<0.0001
Baseline GFAP [pg/ml] (median, IQR)	52/52	121.0 (98.6–159.8)	237/237	75.4 (58.3–97.3)	123/123	77.0 (60.1–95.5)	<0.0001
Adjusted baseline GFAP [z-score] (mean, std)	52/52	1.50 (0.85)	236/237	0.68 (1.00)	123/123	0.69 (0.87)	<0.0001

The difference between the groups was statistically significant (*p* = 0.0016), with the only significant difference in the post-hoc testing being the one between depression and AN (*p* = 0.0038). The sex distribution varied from 51 (98.0%) females in the AN group and 186 (78.6%) in the depression cohort to 51 (41.5%) for patients with other psychiatric disorders and was also significantly different across the groups (*p* < 0.0001). Post-hoc analyses showed significant differences between other psychiatric disorders and both depression and AN (both *p* < 0.0001), as well as between depression and AN (*p* = 0.0052). The baseline BMI-SDS (mean (SD)) was −2.90 (1.2) for the AN group, 0.22 (1.45) for the depression cohort, and 0.08 (1.52) for patients with other psychiatric disorders, with a statistically significant difference between groups (*p* < 0.0001). In this case, post-hoc analyses only showed a significance between AN patients and both other groups (both *p* < 0.0001), but not between the latter groups (*p* = 0.7970).

### sNfL and GFAP in sera are elevated in AN

**Fig. 1:**
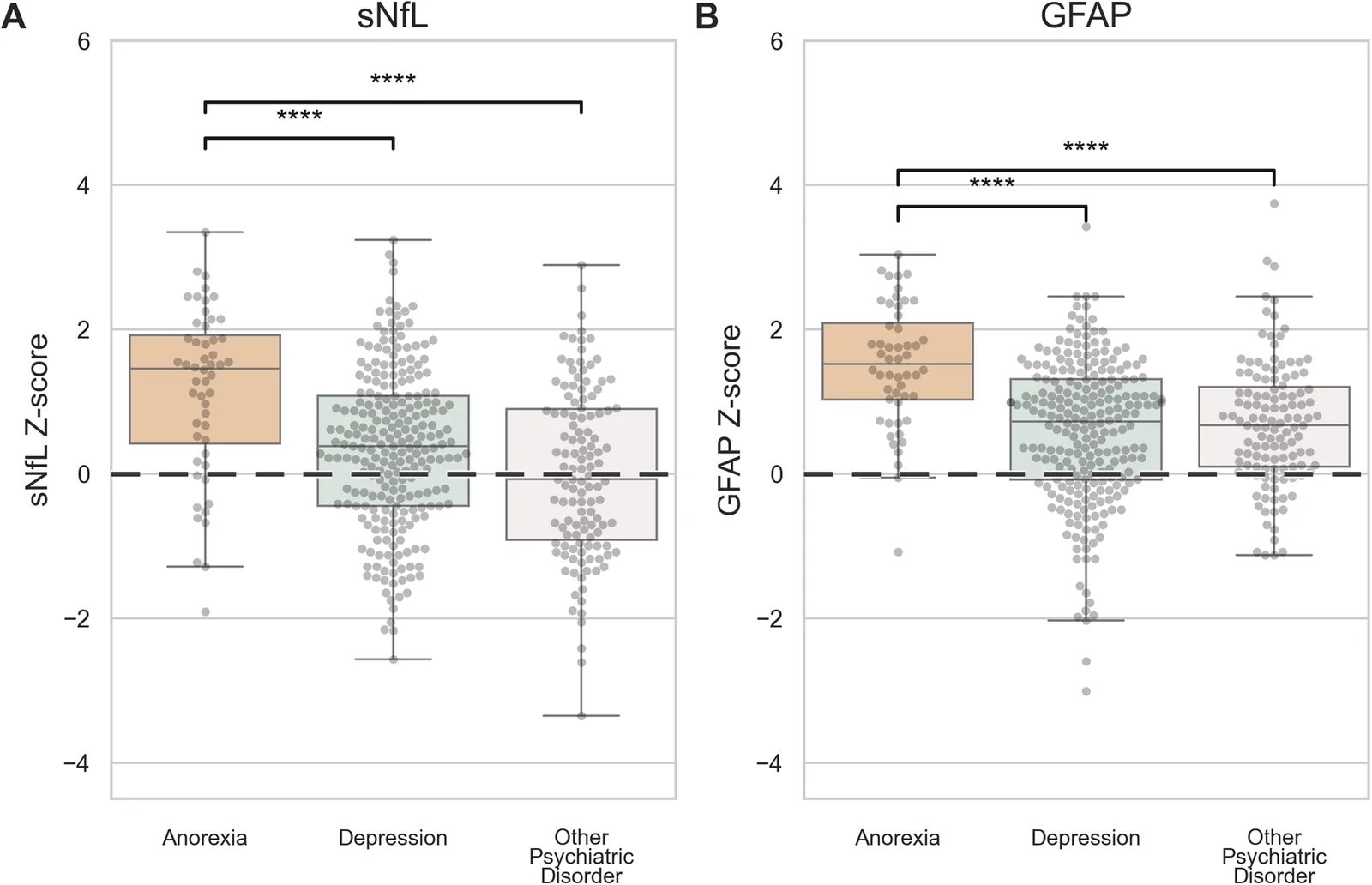
sNfL and GFAP Z-scores Across Pediatric Psychiatric Disease Groups. **A** sNfL Z-scores are shown using combined boxplots and swarm plots for patients with anorexia nervosa (AN, orange), depression (green), and other psychiatric disorders (light pink). AN patients exhibit significantly higher sNfL values compared to the other groups. **B** Similarly, GFAP Z-scores are presented for the same patient groups, with AN patients again showing significantly higher values. The boxplot center line represents the median, the box limits correspond to the upper and lower quartiles, and the whiskers indicate 1.5 times the interquartile range. Each grey point represents an individual patient’s value. Post-hoc pairwise comparisons were calculated using Tukey’s Honest Significant Difference tests. Significant results from Tukey’s test are indicated with asterisks above the relevant comparisons. ANOVA results: (**A**) sNfL, F(2,409) = 18.56 F(2, 409) = 18.56 F(2,409) = 18.56, *p* < 0.0001; (**B**) GFAP, F(2,408) = 20.36 F(2, 408) = 20.36 F(2,408) = 20.36, *p* < 0.0001. AN anorexia nervosa, ANOVA analysis of variance, GFAP glial fibrillary acidic protein, sNfL serum neurofilament light chain. ****P < 0.0001.

Figure 1 illustrates significantly higher Z-scores of sNfL (Fig. 1A) and GFAP (Fig. 1B) in patients with AN compared to those with either depression or other psychiatric disorders. The sNfL Z-score for AN patients with a mean (SD) of 1.15 (1.17) was notably higher than that of depression patients (0.34 (1.10), *p* < 0.0001) and patients with other psychiatric disorders (0.01 (1.17), *p* < 0.0001). Between the latter groups no statistically significant difference could be observed. Similar patterns of differences were observed in GFAP Z-scores with a mean of 1.50 (0.84) for AN compared to 0.58 (1.00) (*p* < 0.0001) for depression and 0.69 (0.87) (*p* < 0.0001) for other psychiatric disorders, with again no significant difference between the latter two. The analysis of raw sNfL and GFAP values showed an identical pattern (Supplementary Fig. 2). When tested against the reference population with a Z-score of 0 (±1 SD), patients with AN (*p* < 0.0001) and depression (*p* < 0.0001) showed statistically higher sNfL values compared to the reference population, while those with other psychiatric disorders did not differ. In case of GFAP Z-scores, all three patient cohorts showed significantly higher values compared to the underlying reference population (each *p* < 0∙0001). Within the group of other psychiatric disorders, a comparison between internalizing and externalizing presentations did not indicate significant heterogeneity for either biomarker (sNfL *p* = 0.488; GFAP *p* = 0.565). An exploratory comparison across the primary subgroups (i.e. patients with anxiety or phobia, attention-deficit/hyperactivity, conduct disorder or cannabis dependence) was likewise not significant (sNfL *p* = 0.463 for general differences; GFAP *p* = 0.897 for general differences).

### sNfL and GFAP – sensitivity analyses: BMI

As BMI is a known effect modifier of sNfL and GFAP levels we first examined its effect on both across our adolescent psychiatric cohort. As shown in Supplementary Table 1 linear regression models revealed negative associations between BMI-SDS and raw sNfL levels across the complete sample (β = −1.08, R² = 0.19, *p* < 0.0001). When analyzing diagnostic groups separately, this was confirmed for patients with depression (β = −0.78, R² = 0.13, *p* < 0.0001) and other psychiatric disorders (β = −0.39, R² = 0.07, *p* = 0.0113), but, interestingly, not for AN (*p* = 0.6254). For GFAP, a similar negative association was observed across the entire cohort (β = −8.77, R² = 0.11, *p* < 0.0001), however, on the subgroup level this was only confirmed for depression (β = −6.13, R² = 0.06, *p* = 0.0005) and not for AN (*p* > 0.99) or other psychiatric disorders (*p* = 0.9493) (Supplementary Table 2).

**Fig. 2:**
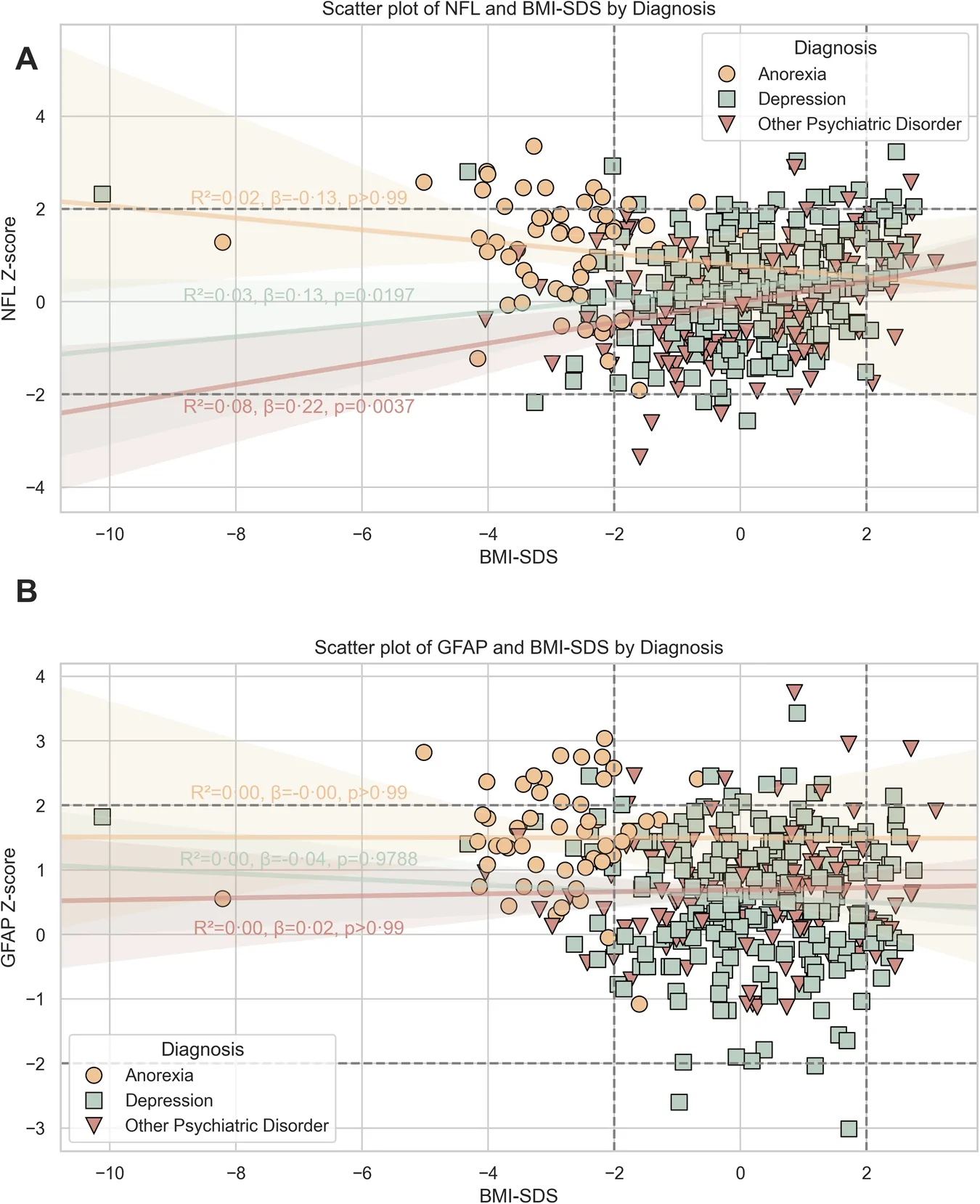
Influence of BDI SDS Z-score on sNfL and GFAP Z-scores by Diagnosis. **A** The scatterplot shows the relationship between BMI SDS Z-scores (x-axis) and sNfL Z-scores (y-axis) across different diagnosis groups. Anorexia nervosa (AN) patients are represented by orange circles, depression patients by turquoise squares, and other psychiatric patients by red triangles. The lines represent linear regressions for each group, with the corresponding R² values indicating the proportion of variance explained by the model: AN β = −0.13, R² = 0.02 (*p* > 0.99), depression β = 0.13, R² = 0.03 (*p* = 0.020), and other Psychiatric Disorders β 0.22, R² = 0.08 (*p* = 0.004). **B** Similarly, the scatterplot shows the relationship between BMI SDS Z-scores and GFAP Z-scores. The R² values of all relationships are 0.00 with all *p*-values above 0.05. All *p*-values are reported Bonferroni-corrected within the respective biomarker group. AN anorexia nervosa, BMI SDS body mass index standard deviation score, sNfL serum neurofilament light chain, GFAP glial fibrillary acidic protein.

Subsequently, we tested whether these biases persisted when Z-scores were used. Figure 2 shows different trends for the relationship between BMI-SDS and Z-score transformed sNfL and GFAP for each diagnosis group. Linear regression analyses showed positive associations between BMI-SDS and sNfL Z-scores in the group of depressed (β = 0.13, R² = 0.03, *p* = 0.0197) and other psychiatric patients (β = 0.22, R² = 0.08, *p* = 0.0037), meaning that BMI effects on sNfL were not fully removed in these two groups by calculating Z-scores using data from a reference group. A relationship for patients with AN (β = −0.12, R² = 0.02, *p* > 0.99) was, as with the raw values before, not significant (Supplementary Table 3). For the GFAP Z-scores and BMI-SDS no significant relationships were identified in any subgroup (Supplementary Table 4).

**Fig. 3:**
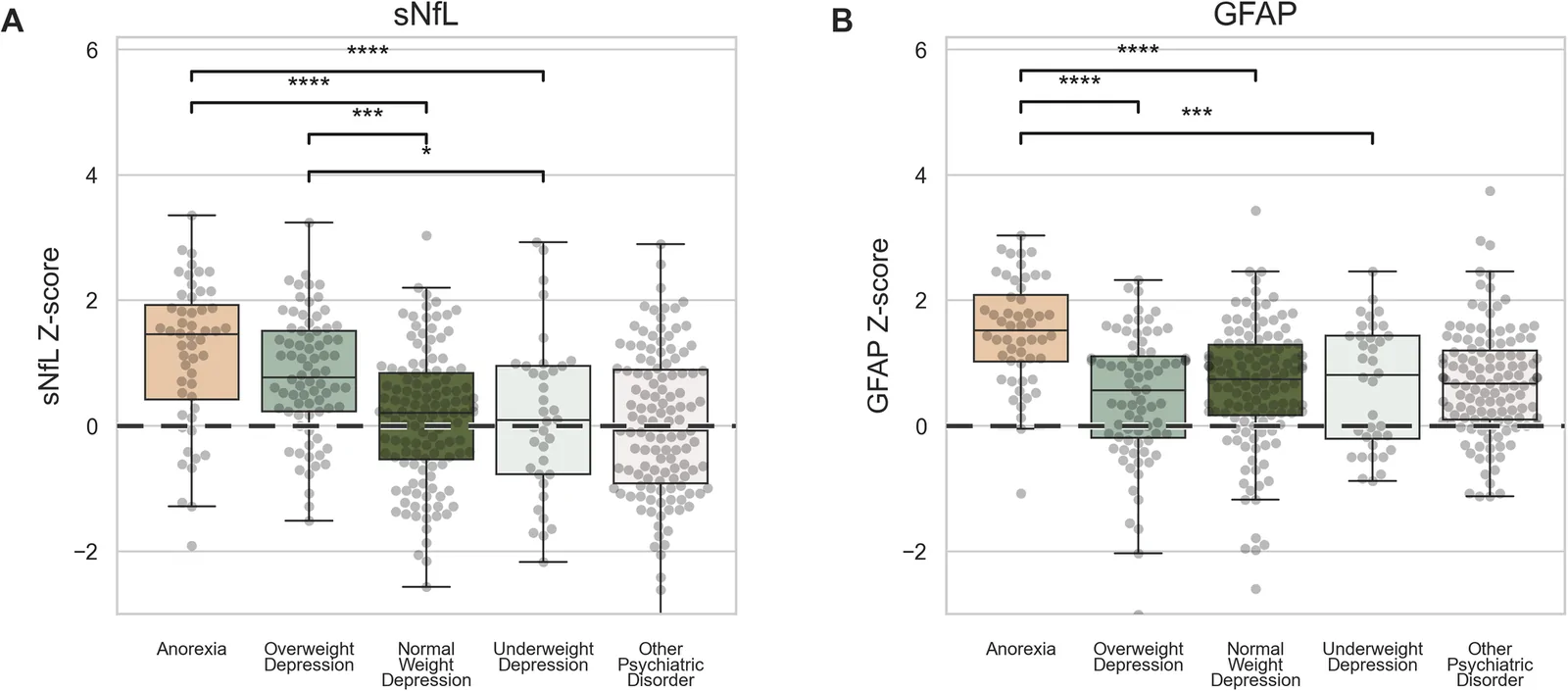
sNfL and GFAP Z-scores for Anorexia Nervosa and Depression Patients by Weight Categories. **A** The combined boxplot and swarmplot show sNfL Z-scores for anorexia nervosa patients (AN, orange) and depression patients stratified by weight categories: overweight (turquoise), normal weight (green), and underweight (light gray). Patients with AN show significantly higher sNfL Z-scores compared to normal and underweight depression patients. Additionally, overweight depression patients exhibit higher sNfL Z-scores than normal and underweight depression patients. **B** GFAP Z-scores are presented for the same patient groups, with AN patients having significantly higher GFAP Z-scores compared to all depression patient weight categories. The boxplot center line represents the median, the box limits correspond to the upper and lower quartiles, and the whiskers indicate 1.5 times the interquartile range. Each grey point represents an individual patient’s value. Post-hoc pairwise comparisons were calculated using Tukey’s Honest Significant Difference tests. Significant results from Tukey’s test are indicated with asterisks above the relevant comparisons. ANOVA results: (**A**) sNfL, F(3,284) = 14.44 F(3, 284), *p* < 0.001p; (**B**) GFAP, F(3,284) = 13.20 F(3, 284), *p* < 0.0001. AN anorexia nervosa, ANOVA analysis of variance, GFAP glial fibrillary acidic protein, sNfL serum neurofilament light chain. ***p < 0.001; ****p < 0.0001.

Considering that not only AN, but also depression can result in substantial underweight and also considering the potential residual BMI bias described above, we further divided the group of patients with depression into normal-weight (*n* = 124), overweight (*n* = 76), and underweight (*n* = 36) subgroups (one patient lacked a BMI-SDS-based weight classification) and compared these with AN patients. Comparing these stratified groups, overweight patients showed higher sNfL Z-scores (mean (SD) 0.80 (0.96)) compared to both normal-weight (0.13 (1.05), *p* = 0.0002) and underweight patients (0.13 (1.27), *p* = 0.0145) with depression (Fig. 3A). While AN (1.15 (1.17)) patients showed significantly higher sNfL Z- scores than underweight (0.14 (1.28)) and normal weight depression patients (0.13 (1.05), *p* ≤ 0.0001 for both), there was no significant difference compared to patients with concurrent overweight and depression (0.80 (0.97), *p* = 0.2709). The AN-versus-underweight depression difference in sNfL Z-scores corresponded to a large standardized effect (Cohen’s d ≈ 0.83). When looking at raw sNfL values instead of Z-scores, AN showed significantly higher values than all depression patient groups stratified by weight status (to normal and overweight: *p* < 0.0001, to underweight: *p* = 0.0031) Supplementary Fig. 3A), with overweight depression patients depicting significantly lower levels than the other weight groups. Antidepressant use was reported separately (19.1%) but was also part of the broader psychopharmacological medication category (22.9%). Neither antidepressant use nor overall psychopharmacological medication use differed significantly across underweight, normal-weight, and overweight depression subgroups (antidepressants *p* = 0.865; any psychopharmacological medication *p* = 0.726). After adjustment for age, sex, and weight group, neither variable was associated with baseline sNfL or GFAP Z-scores (all *p* > = 0.534), and their inclusion only minimally changed the depression weight-group coefficients (delta R² < = 0.002).

GFAP Z-scores in patients with AN were significantly higher in comparison to all other depression subgroups (*p* < 0.0001 for normal and overweight, *p* = 0.0004 for underweight) without significant differences among these subgroups (Fig. 3B). In this case, the AN-versus-underweight depression difference in GFAP Z-scores corresponded to a large standardized effect (Cohen’s d ≈ 0.95). Using raw GFAP values confirmed highest values in patients with AN, while results were slightly different in patients with depression. Here, underweight patients with depression had significantly higher values than overweight patients with depression (*p* = 0.0059) (Supplementary Fig. 3B). Internal normalization using an all-female adolescent control group (*n* = 25, age in years [IQR]: 17.15 [15.30, 17.94], BMI-SDS [IQR]: −0.48 [−0.84, 0.21]) attenuated coefficient magnitudes on the SD scale but did not materially alter the principal diagnosis-versus-control pattern: AN remained the numerically strongest signal for both biomarkers, depression remained significantly elevated versus controls, and all main diagnosis-versus-control contrasts remained positive and statistically significant after internal normalization (see Supplementary Tables 5 and 6).

#### Exploratory ROC analyses of cross-diagnostic biomarker separation

**Fig. 4:**
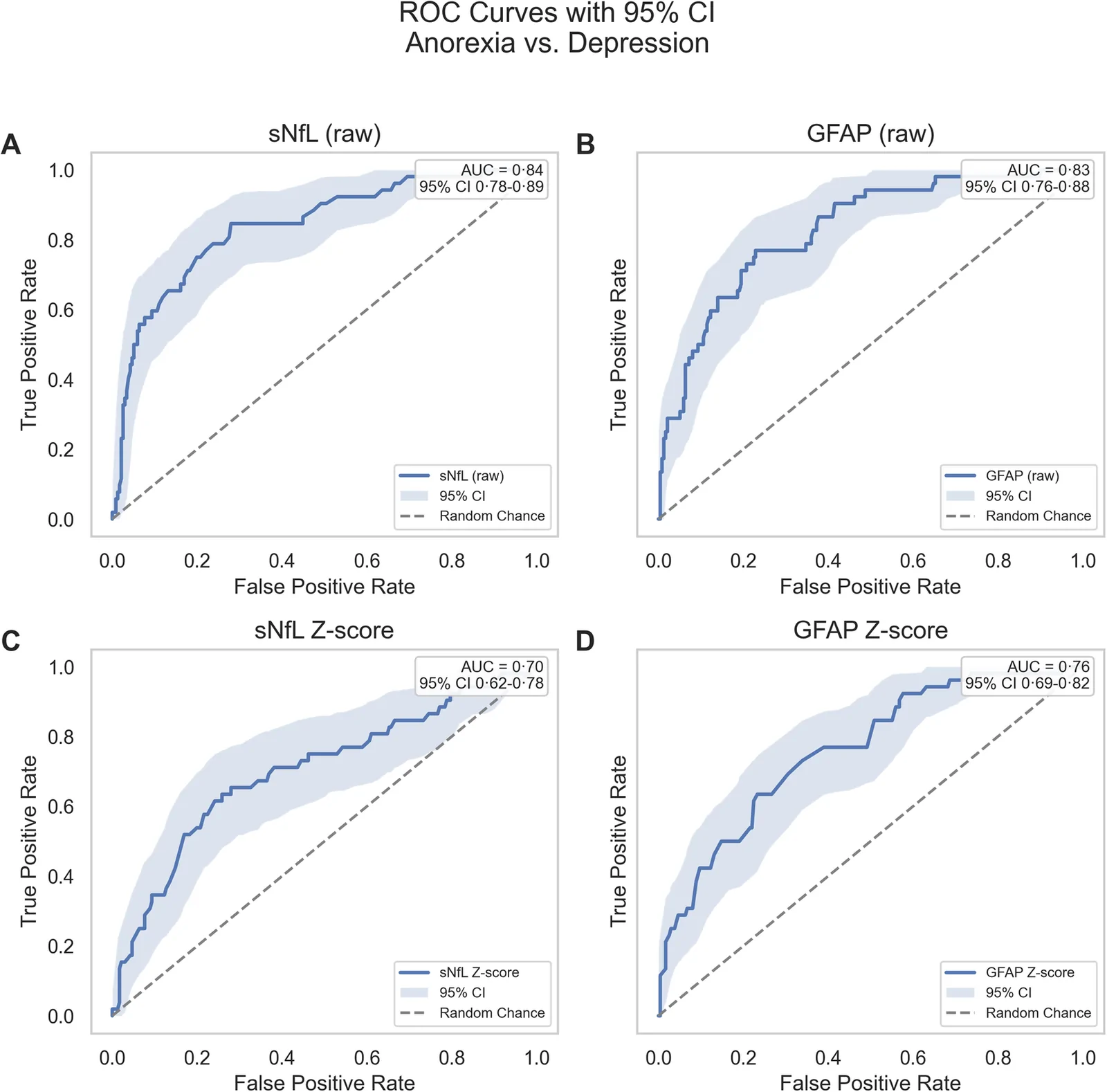
ROC Analysis of Serum Biomarkers Distinguishing Anorexia Nervosa from Depression. Receiver Operating Characteristic (ROC) curves with 95% confidence intervals (CIs) for distinguishing anorexia nervosa (AN) from depression based on sNfL and GFAP measurements. Shaded areas indicate the 95% CIs derived from 1000 bootstrap resamples. All reported thresholds and performance metrics (sensitivity, specificity) reflect the Youden’s J criterion. **A** sNfL (raw) shows an AUC of 0.84 (95% CI [0.779, 0.900]) with a mean threshold of 6.905 (95% CI [6.200, 9.590]), mean sensitivity of 0.794 (95% CI [0.596, 0.923]), mean specificity of 0.790 (95% CI [0.678, 0.941]), and mean Youden’s J of 0.584 (95% CI [0.476, 0.691]). **B** GFAP (raw) yields an AUC of 0.83 (95% CI [0.762, 0.882]) with a mean threshold of 99.224 (95% CI [78.500, 116.000]), mean sensitivity of 0.786 (95% CI [0.615, 0.962]), mean specificity of 0.767 (95% CI [0.555, 0.898]), and mean Youden’s J of 0.553 (95% CI [0.435, 0.672]). **C** sNfL Z-score has an AUC of 0.70 (95% CI [0.617, 0.784]) with a mean threshold of 1.113 (95% CI [0.674, 1.514]), mean sensitivity of 0.643 (95% CI [0.481, 0.808]), mean specificity of 0.753 (95% CI [0.589, 0.864]), and mean Youden’s J of 0.397 (95% CI [0.266, 0.534]). **D** GFAP Z-score exhibits an AUC of 0.76 (95% CI [0.686, 0.828]) with a mean threshold of 1.182 (95% CI [0.412, 1.751]), mean sensitivity of 0.720 (95% CI [0.500, 0.962]), mean specificity of 0.708 (95% CI [0.419, 0.911]), and mean Youden’s J of 0.428 (95% CI [0.311, 0.557]). For details see Supplementary Table 7. AN anorexia nervosa, AUC area under the curve, CI confidence interval, GFAP glial fibrillary acidic protein, ROC receiver operating characteristic, sNfL serum neurofilament light chain, Youden’s J Youden’s index.

To contextualize whether the AN-associated biomarker signal was distinguishable from depression, we performed exploratory bootstrapped ROC analyses. Despite the limited sample size, exploratory bootstrapped ROC analyses suggest sNfL and GFAP may help differentiate AN from depression. This tendency was observed across the analyzed biomarkers, with raw values showing higher AUCs on average. Mean AUCs ranged from 0.84 (95% CI: 0.779–0.900) for raw sNfL and 0.83 (95% CI: 0.762–0.882) for raw GFAP to 0.70 (95% CI: 0.617–0.784) and 0.76 (95% CI: 0.686–0.828) for Z-score transformed sNfL and GFAP, respectively (Fig. 4). Since raw values were shown to correlate with BMI, potentially reflecting diagnostic group differences, we recalculated AUCs to better differentiate AN from underweight depression patients, thereby reducing BMI-related effects. Following this adjustment, AUCs for both raw and Z-score-transformed serum biomarkers ranged from 0.73 to 0.75 (Supplementary Fig. 4). These AUCs fall within the moderate to very good range reported in the literature [23]. Further biomarker statistics, including Youden’s J, the calculated thresholds, and the corresponding sensitivity and specificity, can be found in Supplementary Table 7.

#### Association of sNfL and GFAP with clinical disease stage

Associations with clinical severity were tested within AN and depression groups. No significant relationships were found between BDI-II scores in depression patients and either serum biomarker (*p* > 0.99, respectively). In AN, T0 sNfL Z-scores showed a significant association with the difference between premorbid BMI-SDS and BMI-SDS at T0 (β = −0.45, R² = 0.20, *p* = 0.0248), meaning that greater weight loss in AN patients are linked to higher sNfL Z-scores (Supplementary Fig. 5A). This represented a moderate effect size, explaining approximately 20% of the variance in baseline sNfL Z-scores. For GFAP, no significant relation with BMI-SDS was observed (β = −0.08, R² 0.01, *p* > 0.99) (Supplementary Fig. 5B).

**Fig. 5:**
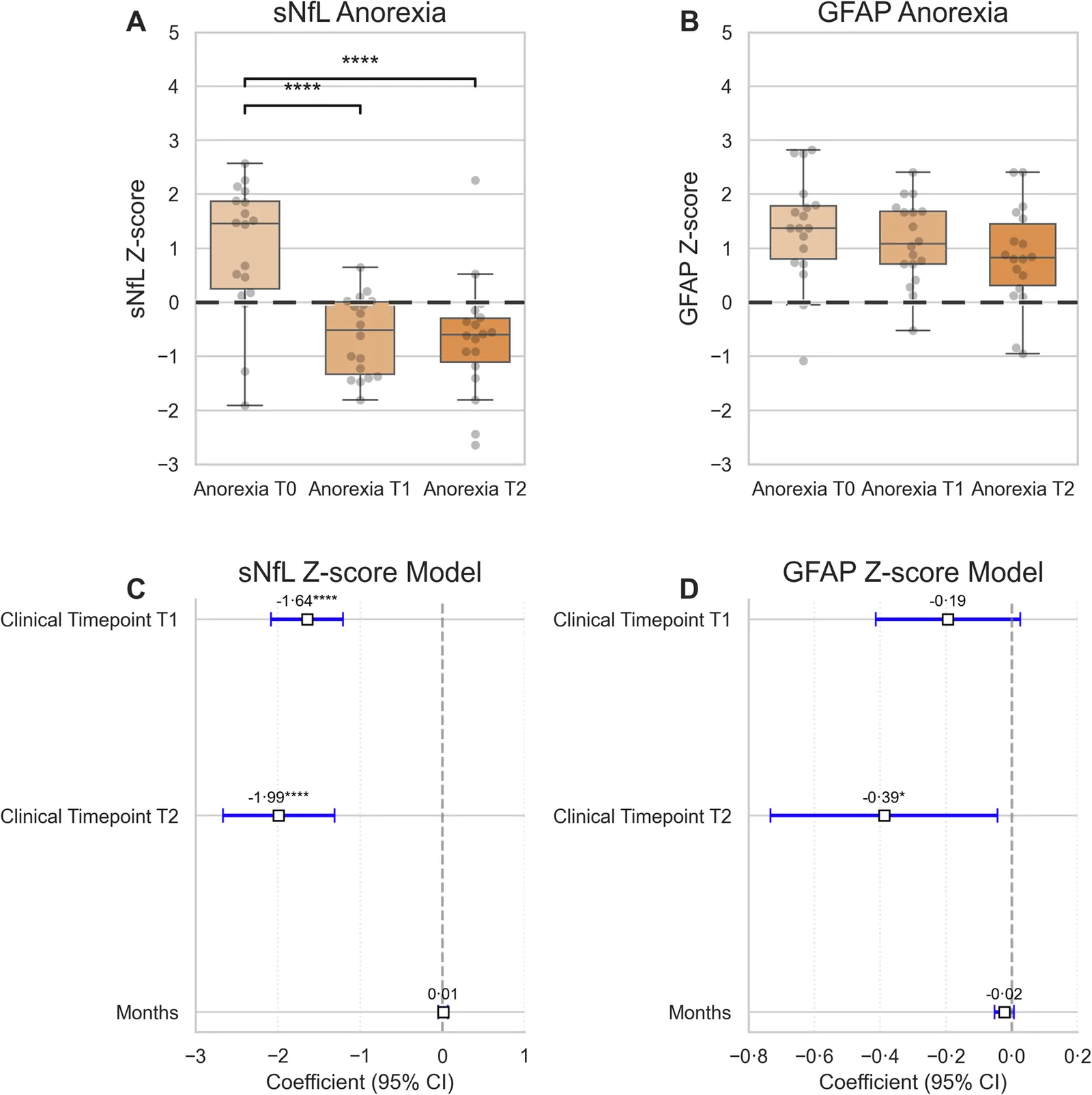
Development of sNfL and GFAP Z-Scores by Clinical Stage in Pediatric Anorexia Patients. **A,** **B** Development of sNfL and GFAP Z-scores in pediatric anorexia nervosa (AN) patients across three clinical timepoints - T0 (admission), T1 (half of target weight), and T2 (long-term weight regain and close to target weight). Median time to follow up was a median of 55.0 (49.0–61.0) days for T1 and 139.0 (92.0–393.0) for T2. Only individuals with measurements across all timepoints were included in this analysis (*n* = 17). Boxplots depict median, upper and lower quartiles, and 1.5× interquartile range whiskers, with grey dots represent individual participants. Post-hoc Tukey’s tests indicate significantly higher sNfL Z-scores at T0 vs. T1 and T2. For GFAP, no significant differences were observed in the post-hoc comparisons. Repeated Measures ANOVA results: (**A**) sNfL, F(2, 34) = 27.41, *p* < 0.001; (**B**) GFAP, F(2, 34) = 6.02, *p* = 0.006. **C,** **D** In a second approach, a mixed-effects model was fit on data from all AN patients (*n* = 52) with all measurements (T0, *N* = 52; T1, *N* = 26; T2, *N* = 22). Forest plots from the mixed-effects model (“Serum Biomarker ~ Elapsed months since admission + Clinical Timepoint + (1|Patient_ID)”). Here, elapsed time denotes months since admission derived from the exact sampling dates. Each square marker shows the coefficient estimate; horizontal lines indicate the 95% CI. Numerical coefficient values appear above the markers, accompanied by stars reflecting significance thresholds. In the sNfL model, negative coefficients for T1 and T2 indicate lower sNfL levels compared to T0, with no significant effect of elapsed time (“Months”) alone. For GFAP, only T2 had a significant negative coefficient, which was smaller than the coefficients observed for sNfL. Details of the models are available in Supplementary Tables 8 and 9. Overall, both approaches congruently illustrate the relative impact of clinical timepoints on serum biomarkers in adolescent AN, with GFAP showing more modest changes across time. ***p < 0.001; ****p < 0.0001. AN anorexia nervosa, ANOVA analysis of variance, GFAP glial fibrillary acidic protein, sNfL serum neurofilament light chain.

In a longitudinal analysis of patients with AN, follow-up measurements at T1 and T2 were used as clinical proxies for therapeutic response. For descriptive readability, time to follow-up is reported in days after admission (T1 half-target weight, median [IQR]: 55 [49–61] days after T0 and T2 near-target weight, median [IQR]: 139 [92–393] after T0 or 67 [40–333] days after T1), whereas the mixed-effects model used elapsed months since admission derived from the exact sampling dates. SNfL and GFAP measurements were strongly correlated at baseline, with baseline GFAP showing stronger correlations with subsequent measurements over time than sNfL (Supplementary Fig. 6). Additionally, when considering only patients with measurements at all timepoints, the elevated sNfL value at T0 decreased and normalized by T1 and T2 (each *p* < 0.0001), without significant difference between both latter timepoints. In contrast, GFAP levels also decreased over time, but these differences were not statistically significant after *p*-value adjustment (Fig. 5A and B). These findings were generally consistent when examining raw concentrations over time (Supplementary Fig. 7). Building upon these observations, we next fitted a linear mixed-effects model to predict serum biomarkers from clinical timepoints and elapsed time, including all available measurements, even those missing one or more timepoints. Consistent with the repeated-measures analysis, clinical timepoints T1 (β = −1.64, *p* < 0.0001) and T2 (β = −1.99, *p* < 0.0001) both significantly reduced sNfL relative to T0, whereas elapsed months since admission did not exert a meaningful influence (*p* = 0.75) (Fig. 5C). In contrast, only T2 (β = −0.39, *p* = 0.028) reached significance in the GFAP model, indicating a more modest overall effect (Fig. 5D). Complete model statistics, including random intercept estimates, are presented in Supplementary Tables 8 and 9.

## Discussion

Research on psychiatric serum biomarkers in youth, especially longitudinal data, is limited. Our study suggests that sNfL and GFAP capture neurobiological stress most prominently in adolescent AN, with sNfL additionally relating to starvation severity and showing longitudinal decline during treatment. In contrast, biomarker elevations in depression were less distinct and were not associated with depressive symptom severity. Thus, the present findings primarily support these markers as translational and longitudinal indicators of AN-related biological burden. To a more limited degree, this study indicates that sNfL and GFAP may have potential as supplemental diagnostic tool for AN e.g. in contrast to underweight patients with depression, with higher levels than a healthy reference and other disorders, correlating with symptom severity.

Previous studies on psychiatric biomarkers, mainly in adults, have yielded inconsistent evidence of elevated sNfL or GFAP levels, limiting their clinical applicability [15, 16]. In adolescents, research has been further hindered by small sample sizes, inadequate cross-disorder comparisons, inclusion of comorbidities, and unaddressed confounding factors [16, 24]. Accurate interpretation of sNfL and GFAP measurements requires understanding factors affecting their precision, such as body weight. BMI- and age-adjusted Z-scores (including sex for GFAP) have been established for adults [9, 21]. Recently published reference values for sNfL in adolescents considered age, but not BMI [9]. We are the first to investigate both markers in adolescents with psychiatric disorders while considering confounders. AN patients showed significantly higher biomarker levels than underweight depression patients, regardless of measurement modality (raw concentrations or Z-scores). Notably, the comparison with underweight depression showed large standardized differences for both biomarkers (sNfL Z-score Cohen’s d ≈ 0.83; GFAP Z-score Cohen’s d ≈ 0.95), supporting that the observed contrasts were not only statistically significant but also clinically meaningful in magnitude. While raw values were higher in underweight than overweight depression patients [11, 16], BMI-adjusted Z-scores showed the opposite trend. This highlights the importance of considering BMI when interpreting sNfL, especially given the observed correlation between BMI and both sNfL and GFAP in this adolescent population. A limitation is that the BMI Z-standardized data may not accurately reflect the extreme BMI values in the AN cohort due to extrapolation. However, lower BMI in the reference sample was also associated with lower sNfL levels [9]. This approach confirmed significantly elevated sNfL and GFAP in pediatric AN compared to both a reference group and patients with depression or other psychiatric disorders. While biomarkers were also elevated in depression, they lacked diagnostic specificity and correlation with severity. Exploratory AUC analyses showed moderate to very good discrimination of AN from depression and underweight depression, highlighting their potential in complex cases. In practical terms, the observed AUCs suggest clinically relevant but not definitive discrimination. Thus, sNfL and GFAP may be most useful as adjunctive biomarkers in challenging differential diagnoses rather than as standalone diagnostic tests. A reasonable interpretation of the AUC analyses is that the discriminatory signal is partly BMI-driven, especially for raw biomarker values, but not reducible to BMI alone: AN remained separable from underweight depression, GFAP Z-scores showed no residual BMI effect, and the residual BMI associations for sNfL Z-scores were small and not present in AN, supporting an additional disease-related component of biomarker elevation. Because ROC estimates involving the AN group were based on a comparatively small sample (*n* = 52), these analyses should be regarded as exploratory and potentially less stable than those derived from more balanced group comparisons.

In our study, we found no significant changes in sNfL and GFAP levels across other psychiatric disorders, though the Z-score for GFAP was slightly increased when compared to the reference population. Consequently, there is currently no evidence of consistent alterations in either biomarker across these conditions [16, 25], particularly with AN and depression excluded as comorbidities. In addition, emerging evidence suggests that biomarker elevations in psychiatric cohorts may in part reflect acute illness burden or substance-related neurobiological stress rather than disorder-specific effects [25, 26].

Our findings suggest neuroaxonal and astrocytic damage in AN, with elevated sNfL and GFAP correlating with MRI-detected gray and reversible white matter changes during weight restoration [24, 27]. MRI provides insights into psychiatric disorders [5] but is challenging to integrate clinically, especially in adolescents. sNfL and GFAP may offer a standardized blood-based alternative for assessing structural brain changes.

*Hellerhoff et al*. found elevated sNfL and GFAP levels in underweight AN patients, which decreased after partial weight restoration [24]. Similarly, our study linked weight loss, as a proxy for starvation severity, to higher sNfL levels. This suggests sNfL may help assess disease severity, particularly at onset, and reflect therapeutic progress through weight normalization. The faster normalization of sNfL compared with GFAP is in line with investigations showing sNfL peaks approximately one month after damage with normalization over few months [28]. Our study, however, further highlights, that rather than the elapsed time, it appears that the clinical improvement of the patient is predictive of sNfL (and to a lesser degree GFAP) levels, warranting further investigation into its potential as longitudinal biomarker.

In line with the known kinetics of sNfL, which typically reflect dynamic neuroaxonal injury and recovery, this supports the interpretation that biomarker normalization represents resolution of starvation-related neurobiological stress rather than a purely time-dependent process.

From a clinical perspective, translating these findings into practical application remains an important next step. Based on our data, sNfL and GFAP may be most relevant in three preliminary contexts. First, in diagnostically challenging cases, such as underweight adolescents with abnormal eating behaviour and depressive symptoms, elevated sNfL and GFAP may support the presence of starvation-related neurobiological involvement, complementing clinical assessment, weight status, starvation-related symptoms, and biochemical parameters such as leptin [29]. However, it remains unclear at which stage of illness development these biomarkers become detectable and how they relate to emerging psychopathology, weight loss, and somatic consequences of starvation.

Second, in established AN, sNfL in particular may serve as a longitudinal marker of disease severity and treatment response, as levels declined consistently with weight restoration. Persistently elevated sNfL despite apparent clinical or behavioural improvement could indicate ongoing neurobiological stress and may warrant closer monitoring. Third, both biomarkers may currently be best positioned as translational tools to better characterize the individual extent of brain involvement in AN and related psychiatric conditions, rather than as standalone clinical decision-makers. Future studies should clarify their relationship with clinical, biochemical, and brain-related markers, including hormones and neurotrophic factors [29, 30]. Ultimately, multidimensional approaches may help integrate such information into clinically useful prediction models.

While formal clinical thresholds cannot be derived from the present data, our findings suggest that sNfL Z-scores above the population mean, particularly values exceeding approximately 1 standard deviation as observed in a substantial proportion of patients with AN, may reflect clinically relevant neuroaxonal stress. Prospective studies are needed to establish actionable cut-offs, define meaningful longitudinal changes, and determine whether biomarker-guided approaches, for example as part of composite scores integrating multiple relevant markers, can improve patient outcomes.

One limitation of our study is the heterogeneous composition *of the group with “other psychiatric disorders”*, which included a broad range of diagnoses. This group was not designed to allow disorder-specific conclusions but rather to provide a pragmatic comparator for non-AN, non-depression psychiatric conditions. Accordingly, the absence of significant biomarker differences in this group should be interpreted with caution, as potential effects within individual diagnostic subgroups may have been obscured by heterogeneity and limited subgroup sizes. Additionally, the lack of longitudinal depression data hinders biomarker dynamics assessment. Follow-ups are less standardized than treatment milestones, and early clinical improvements may precede detectable sNfL and GFAP changes [31]. The usage of adult reference values in an adolescent sample represents a further limitation of our study. Since current pediatric cohorts lack proper BMI and age adjustments which would be necessary for a more precision estimation of effects, future research should incorporate these factors to improve Z-score accuracy and reduce potential overcorrection. Nevertheless, we conducted a further sensitivity analysis using internally normalized raw biomarker values which yielded a materially consistent diagnosis-wise pattern, arguing against the principal findings being explained by the adult reference datasets alone. Applying adult reference data which are truncated regarding lower BMI levels may have caused a modest upward bias of Z-scores in younger adolescents and at the extreme low-BMI tail. However, the main diagnosis-level findings were also seen in raw values and after internal normalization, suggesting that this limitation mainly affects finer subgroup contrasts rather than the overall AN-related signal. Since information on substance abuse (i.e. alcohol, nicotine, and cannabis) was not available for all participants, adjustment for these factors in our statistical analysis was not possible. Accordingly, residual confounding cannot be ruled out.

In conclusion, our study supports sNfL and GFAP as biomarkers of AN-related neurobiological stress in adolescent psychiatric populations. The persistence of group differences in comparisons with underweight depression suggests that these findings reflect more than low body weight alone. sNfL in particular may be useful as a longitudinal marker of starvation-related disease burden and treatment response, while both biomarkers may serve as translational tools for studying biologically relevant dimensions of adolescent psychopathology.

## Supplementary information

Supplemental material

## Data Availability

Pseudonymised biomarker data and associated clinical variables underlying the main analyses will be shared upon reasonable request, subject to a data-sharing agreement, ethics approval where applicable, and compliance with data protection regulations.
