# Supplementary material for: Exploring serum biomarkers in paediatric psychiatry: the impact of NfL and GFAP
Source: Transl Psychiatry. 2026 Aug 26;16:431. doi: 10.1038/s41398-026-04347-1 (PMC13518902; doi:10.1038/s41398-026-04347-1)
Supplement: Supplementary file 1 [file 41398_2026_4347_MOESM1_ESM.pdf]

Contents

Supplemental Methods 2
Supplemental Table 1: Regression Results (Raw sNfL) 5
Supplemental Table 2: Regression Results (Raw GFAP) 6
Supplemental Table 3: Regression Results (sNfL Z-score) 7
Supplemental Table 4: Regression Results (GFAP Z-score) 8
Supplemental Table 5: Diagnosis-wise Effects versus Control (Internally Normalized sNfL) 9
Supplemental Table 6: Diagnosis-wise Effects versus Control (Internally Normalized GFAP) 10
Supplemental Table 7: Biomarker Performance for Differentiating Anorexia and (Underweight) Depression 11
Supplemental Table 8: Mixed Effects Model Summary (sNfL) 12
Supplemental Table 9: Mixed Effects Model Summary (GFAP) 13
Supplemental Figure 1: Study and Analysis Overview 14
Supplemental Figure 2: Elevated sNfL and GFAP Concentrations in Pediatric Anorexia Patients 15
Supplemental Figure 3: sNfL and GFAP Concentrations for Anorexia Nervosa and Depression Patients by Weight Categories 16
Supplemental Figure 4: ROC Analysis of Serum Biomarkers Distinguishing Anorexia Nervosa from Underweight Depression 17
Supplemental Figure 5: Relationship between Premorbid BMI-SDS Differences and sNfL or GFAP Z-Scores in Anorexia Nervosa 19
Supplemental Figure 6: Correlation Matrix of sNfL and GFAP Across Clinical Stages in Anorexia Patients 20
Supplemental Figure 7: Development of sNfL and GFAP Concentrations by Clinical Stage in Pediatric Anorexia Patients 21

Supplemental Methods

Study sample

Patients aged 11–18 years were included, excluding those with renal/cardiac failure, intellectual disabilities (IQ < 70), or unclear diagnoses. For overlapping participants between the dietary intervention study and the cross-sectional study, only data from the later study were used.

Multimodal treatment regiment

Inpatients with AN were treated according to a multimodal treatment program including weight restoration, medical observation, cognitive–behavioral therapy, family therapy and nutritional counselling. As suggested in the German guidelines[1,2] from 2019 for diagnostic and treatment of AN 19, meal plans provided 30–40 kcal/kg body weight/day at the beginning of treatment. During later inpatient phases, meal plans ensured a minimum weekly weight gain of 500 g. After discharge, outpatients were not routinely treated in a standardized manner.

Diagnostic Instruments and Questionnaires

Psychiatric diagnoses were determined using the semi-structured interview 'Schedule for Affective Disorders and Schizophrenia for School-Aged Children- Present and Lifetime Version' (K-SADS-PL)[3] according to DSM-IV or clinical assessments (ICD-10) when K-SADS-PL was unavailable. Diagnoses were harmonized across cohorts, grouping participants into AN (without depression), depression (without AN), or other psychiatric disorders (e.g., anxiety or hyperactivity).

Anthropometric assessments

Anthropometric measurements followed a standardized procedure, with weight being recorded after a 12-hour fast in underwear using the same scale, and height measured with the same stadiometer. These measurements were used to calculate BMI. Age- and sex-independent BMI percentiles and standard deviation scores (BMI-SDS) were determined using German reference data [4] and to classify overweight (BMI≥90th percentile or +1·282 SD), obesity (BMI≥97th percentile or +1·881 SD) and underweight (BMI<10th percentile or -1·282 SD). Normalweight was defined as BMI >10th percentile and <90th percentile. As surrogate for the disease severity of AN, the extent of weight loss was calculated by the difference between the premorbid BMI and the BMI at baseline. The premorbid BMI was calculated by the individual's weight before weight loss and height, based on self-or parent reports at day-/inpatient admission onset [5]. Depression severity was determined using the BDI-II, a 21-item self-reported questionnaire that measures depressive symptoms over the past two weeks and is based on DSM-IV criteria for major depressive disorder [6].

sNfL and GFAP measures

Blood samples were obtained at T0 for all three patient groups as well as T1 and T2 for patients with AN from an antecubital vein through a short catheter in Monovettes (Sarstedt, Nümbrecht, Germany) at early morning after overnight fasting. The blood samples were transferred within an hour after blood sampling to the central laboratory

of the University Hospital Essen. Samples were centrifuged at 3000 rpm for 10 min, pipetted and frozen within 60 min of collection at −20 °C. Every 4 weeks the collected samples were moved to a −80 °C freezer, where they remained frozen until the final laboratory measurements. For the current project, serum samples were sent in 2023 to the Department of Neurology at the University of Mainz for sNfL and GFAP quantification. All laboratory technicians performing the measurements were blinded to the clinical data.

Statistical analysis

Normality of metric data was assessed using the Shapiro-Wilk test. Homogeneity of variances was evaluated using Levene's test to ensure the assumptions for ANOVA were met. Based on the test results, either a one-way ANOVA or the Kruskal-Wallis test was used to assess overall differences in descriptive data as well as sNfL and GFAP levels across the diagnosis groups. The same tests were used for the comparison of sNfL and GFAP levels between the group with AN and sub-groups of patients with depression classified as under-, normal- or overweight. General differences in the proportion of males and females between all groups were tested using Chi-Square test.

One-sample t-tests allowed for testing of the measured Z-scores against the reference population and could thus test whether the mean Z-scores significantly differed from the reference population (Z-score of zero, SD of one). P-values were Bonferroni-corrected for two outcomes and three groups.

In addition, receiver operating characteristic (ROC) analyses were conducted using a 1000-fold bootstrap procedure to estimate the area under the curve (AUC) for each biomarker discriminating between AN and depression. For each bootstrap sample, Youden's J statistic (defined as $sensitivity + specificity - 1$) was used to identify the optimal threshold. This process produced distributions of sensitivity, specificity, and Youden's J, from which 95% confidence intervals were subsequently derived.

For the comparison of longitudinal biomarker measurements, correlations between biomarker Z-scores in the longitudinally measured AN sub-cohort were calculated using Pearson correlation coefficients following normality assessment. For these longitudinal analyses, p-values were corrected using the false discovery rate (Benjamini-Hochberg) method, chosen over Bonferroni due to the exploratory nature of the analysis, to balance sensitivity and control of false positives. For longitudinal analyses, T1 and T2 denote clinical recovery stages. Elapsed time for the mixed-effects models was calculated from the exact sampling dates as months since admission, whereas follow-up timing is reported descriptively in days after admission where applicable.

References of Supplemental Methods:

1 Föcker M, Cecil A, Prehn C, Adamski J, Albrecht M, Adams F *et al.* Evaluation of Metabolic Profiles of Patients with Anorexia Nervosa at Inpatient Admission, Short- and Long-Term Weight Regain—Descriptive and Pattern Analysis. *Metabolites* 2020; **11**: 7.

2 Resmark G, Herpertz S, Herpertz-Dahlmann B, Zeeck A. Treatment of Anorexia Nervosa-New Evidence-Based Guidelines. *J Clin Med* 2019; **8**: 153.

3 Kaufman J, Birmaher B, Brent D, Rao U, Flynn C, Moreci P *et al.* Schedule for Affective Disorders and Schizophrenia for School-Age Children-Present and Lifetime Version (K-SADS-PL): initial reliability and validity data. *J Am Acad Child Adolesc Psychiatry* 1997; **36**: 980–8.

4 Kromeyer-Hauschild K. Definition, Anthropometrie und deutsche Referenzwerte für BMI, Körperumfänge, Hautfalten und Fettmasse. In: Wabitsch M, Hebebrand J, Kiess W, Reinehr T, Wiegand S (eds). *Adipositas bei Kindern und Jugendlichen*. Springer Berlin Heidelberg: Berlin, Heidelberg, 2022, pp 3–24.

5 Coners H, Remschmidt H, Hebebrand J. The relationship between premorbid body weight, weight loss, and weight at referral in adolescent patients with anorexia nervosa. *Int J Eat Disord* 1999; **26**: 171–178.

6 Kumar G, Steer RA, Teitelman KB, Villacis L. Effectiveness of Beck Depression Inventory-II subscales in screening for major depressive disorders in adolescent psychiatric inpatients. *Assessment* 2002; **9**: 164–70.

Supplemental Table 1: Regression Results (Raw sNfL)

Diagnosis	Intercept	β	P-Value (Intercept)	P-Value (β)	R^2	P-Value (Intercept, Bonferroni)	P-Value (β, Bonferroni)
**All Patients**	6·027	-1·082	<0·0001	<0·0001	0·1882		
**Depression**	5·896	-0·777	<0·0001	<0·0001	0·1263	<0·0001	<0·0001
**Other Psychiatric Disorder**	5·186	-0·392	<0·0001	0·0038	0·0672	<0·0001	0·0113
**Anorexia**	7·863	-1·198	0·0102	0·2085	0·0315	0·0307	0·6254

This table summarizes the results of linear regression models. Each row represents a model fitted for a specific patient subgroup (or all patients combined). The coefficients indicate the relationship between BMI SDS Z-score and (unadjusted) sNfL levels. P-values are provided for both the intercept and slope terms, with Bonferroni correction applied to account for multiple comparisons between subgroups (to intercept and slopes separately). R^2 represents the proportion of variance of sNFL explained by the model.

BMI SDS: Body Mass Index Standard Deviation Score, sNfL: Serum Neurofilament Light Chain

Supplemental Table 2: Regression Results (Raw GFAP)

Diagnosis	Intercept	β	P-Value (Intercept)	P-Value (β)	R^2	P-Value (Intercept, Bonferroni)	P-Value (β, Bonferroni)
**All Patients**	86·954	-8·768	<0·0001	<0·0001	0·1102		
**Depression**	82·263	-6·128	<0·0001	0·0002	0·0592	<0·0001	0·0005
**Other Psychiatric Disorder**	85·020	-2·999	<0·0001	0·3164	0·0083	<0·0001	0·9493
**Anorexia**	135·017	0·112	<0·0001	0·9859	0·0	<0·0001	>0·99

This table summarizes the results of linear regression models. Each row represents a model fitted for a specific patient subgroup (or all patients combined). The coefficients indicate the relationship between BMI SDS Z-score and (unaltered) GFAP levels. P-values are provided for both the intercept and slope terms, with Bonferroni correction applied to account for multiple comparisons between subgroups (to intercepts and slopes separately). R^2 represents the proportion of variance explained by the model.

BMI SDS: Body Mass Index Standard Deviation Score, GFAP: Glial Fibrillary Acidic Protein

Supplemental Table 3: Regression Results (sNfL Z-score)

Diagnosis	Intercept	β	P-Value (Intercept)	P-Value (β)	R^2	P-Value (Intercept, Bonferroni)	P-Value (β, Bonferroni)
**All Patients**	0·346	-0·003	<0·0001	0·9223	0·0		
**Depression**	0·315	0·134	<0·0001	0·0066	0·0312	0·0001	0·0197
**Other Psychiatric Disorder**	-0·004	0·223	0·9661	0·0012	0·0829	>0·99	0·0037
**Anorexia**	0·780	-0·128	0·073	0·351	0·0174	0·2191	>0·99

This table summarizes the results of linear regression models. Each row represents a model fitted for a specific patient subgroup (or all patients combined). The coefficients indicate the relationship between BMI SDS Z-score and sNfL Z-scores. P-values are provided for both the intercept and slope terms, with Bonferroni correction applied to account for multiple comparisons between subgroups (to intercept and β separately). R^2 represents the proportion of variance explained by the model.

BMI SDS: Body Mass Index Standard Deviation Score; sNfL: Serum Neurofilament Light Chain.

Supplemental Table 4: Regression Results (GFAP Z-score)

Diagnosis	Intercept	Slope	P-Value (Intercept)	P-Value (Slope)	R^2	P-Value (Intercept, Bonferroni)	P-Value (Slope, Bonferroni)
**All Patients**	0·703	-0·111	<0·0001	0·0001	0·0392		
**Depression**	0·586	-0·044	<0·0001	0·3263	0·0041	<0·0001	0·9788
**Other Psychiatric Disorder**	0·693	0·016	<0·0001	0·7609	0·0008	<0·0001	>0·99
**Anorexia**	1·494	-0·002	<0·0001	0·9877	0·0	<0·0001	>0·99

This table summarizes the results of linear regression models. Each row represents a model fitted for a specific patient subgroup (or all patients combined). The coefficients indicate the relationship between BMI SDS Z-score and GFAP Z-scores. P-values are provided for both the intercept and slope terms, with Bonferroni correction applied to account for multiple comparisons between subgroups (to intercept and β separately). R^2 represents the proportion of variance explained by the model.

BMI SDS: Body Mass Index Standard Deviation Score; GFAP: Glial Fibrillary Acidic Protein.

Supplemental Table 5: Diagnosis-wise Effects versus Control (Internally Normalized sNfL)

Diagnosis	n group	n control	Model n	Unit	Estimate (SD units)	95% CI	p-value
**Other Psychiatric Disorder**	51	25	312	SD units	1.5688	[1.14, 2.00]	<0.0001
**Depression**	185	25	312	SD units	1.6035	[1.23, 1.98]	<0.0001
**Anorexia**	51	25	312	SD units	1.9722	[1.55, 2.40]	<0.0001

This table summarizes the primary diagnosis-wise case-control model using internally normalized sNfL values (SD units) in the female analytic cohort. Estimates represent the mean difference versus the control group. The internally normalized biomarker was derived from raw baseline values after log10 transformation and normalization for age and BMI-SDS in the female baseline sample.

BMI SDS: Body Mass Index Standard Deviation Score; sNfL: Serum Neurofilament Light Chain

Supplemental Table 6: Diagnosis-wise Effects versus Control (Internally Normalized GFAP)

Diagnosis	n group	n control	Model n	Unit	Estimate (SD units)	95% CI	p-value
**Other Psychiatric Disorder**	51	25	312	SD units	1.1373	[0.67, 1.61]	<0.0001
**Depression**	185	25	312	SD units	0.9026	[0.49, 1.31]	<0.0001
**Anorexia**	51	25	312	SD units	1.2954	[0.83, 1.77]	<0.0001

This table summarizes the primary diagnosis-wise case-control model using internally normalized GFAP values (SD units) in the female analytic cohort. Estimates represent the mean difference versus the control group. The internally normalized biomarker was derived from raw baseline values after log10 transformation and normalization for age and BMI-SDS in the female baseline sample.

BMI SDS: Body Mass Index Standard Deviation Score; GFAP: Glial Fibrillary Acidic Protein.

Supplemental Table 7: Biomarker Performance for Differentiating Anorexia and (Underweight) Depression

Comparison	Variable	AUC 95% CI	Mean Threshold (95% CI)	Mean Sensitivity (95% CI)	Mean Specificity (95% CI)	Mean Youden's J (95% CI)
**Anorexia vs Depression**	sNfL (Raw)	[0·776, 0·899]	6·905 [6·200, 9·590]	0·794 [0·596, 0·923]	0·790 [0·678, 0·941]	0·584 [0·476, 0·691]
**Anorexia vs Depression**	GFAP (Raw)	[0·762, 0·886]	99·224 [78·500, 116·000]	0·786 [0·615, 0·962]	0·767 [0·555, 0·898]	0·553 [0·435, 0·672]
**Anorexia vs Depression**	sNfL Z-score	[0·614, 0·783]	1·113 [0·674, 1·514]	0·643 [0·481, 0·808]	0·753 [0·589, 0·864]	0·397 [0·266, 0·534]
**Anorexia vs Depression**	GFAP Z-score	[0·684, 0·829]	1·182 [0·412, 1.751]	0·720 [0·500, 0·962]	0·708 [0·419, 0·911]	0·428 [0·311, 0·557]
**Anorexia vs Underweight Depression**	sNfL (Raw)	[0·622, 0·844]	8·077 [6·200, 9·700]	0·671 [0·500, 0·904]	0·826 [0·556, 0·972]	0·498 [0·335, 0·647]
**Anorexia vs Underweight Depression**	GFAP (Raw)	[0·648, 0·844]	95·556 [65·300, 132·100]	0·797 [0.461, 1·000]	0·644 [0·333, 0·944]	0·440 [0·297, 0·592]
**Anorexia vs Underweight Depression**	sNfL Z-score	[0·615, 0·834]	1·046 [0·175, 1·440]	0·649 [0·500, 0·846]	0·849 [0·639, 0·972]	0·498 [0·331, 0·656]
**Anorexia vs Underweight Depression**	GFAP Z-score	[0·624, 0·831]	0·654 [-0·050, 1·774]	0·841 [0·423, 1·000]	0·580 [0·333, 0·944]	0·421 [0·280, 0·571]

This table summarizes the results of diagnostic comparisons between anorexia and two conditions: depression and underweight depression. Each row presents the performance of a biomarker variable (sNfL or GFAP, raw or Z-score). Metrics include AUC (with 95% confidence intervals), mean thresholds, sensitivities, specificities, and Youden's J indices, providing a comprehensive overview of the models' diagnostic accuracy.

BMI SDS: Body Mass Index Standard Deviation Score; GFAP: Glial Fibrillary Acidic Protein; sNfL: Serum Neurofilament Light Chain.

Supplemental Table 8: Mixed Effects Model Summary (sNfL)

Parameter	Coefficient	Std.Err.	z	P>|z|	0.025	0.975
**Intercept**	1.151	0·149	7·736	0·000	0·859	1·442
**Clinical Timepoint T1**	-1·644	0·223	-7·380	0·000	-2·081	-1·208
**Clinical Timepoint T2**	-1·990	0·347	-5·742	0·000	-2·669	-1·310
**Elapsed months since admission**	0·009	0·029	0·317	0·751	-0·048	0·067
**Group Variance**	0·439	0·274				

This table presents the results of a mixed effects model analyzing sNfL Z-score as the dependent variable, predicted by Clinical Timepoint and Months, with random effects grouped by Patient ID (Pat_ID). Elapsed months since admission was calculated from the exact sampling dates. The Clinical Timepoint variable has been dummy coded with the baseline category (T0) implicit. Coefficients and associated statistics (Std.Err., z-value, p-value, and 95% confidence intervals) are shown for each predictor. Group Variance represents the variability attributed to differences between patients. Below are key model statistics:

• Observations: 100
• Groups (Patients): 52
• Min. group size: 1
• Max. group size: 3
• Mean group size: 1·9
• Scale: 0·7110
• Log-Likelihood: -147·4249
• Convergence: Yes

sNfL: Serum Neurofilament Light Chain

Supplemental Table 9: Mixed Effects Model Summary (GFAP)

Parameter	Coefficient	Std.Err.	z	P>|z|	0.025	0.975
**Intercept**	1·498	0·111	13·466	0·000	1·280	1·716
**Clinical Timepoint T1**	-0·194	0·112	-1·730	0·084	-0·414	0·026
**Clinical Timepoint T2**	-0·388	0·176	-2·203	0·028	-0·733	-0·043
**Elapsed months since admission**	-0·023	0·015	-1·493	0·136	-0·052	0·007
**Group Variance**	0·480	0·414				

This table presents the results of a mixed effects model analyzing GFAP Z-score as the dependent variable, predicted by Clinical Timepoint and Months, with random effects grouped by Patient ID (Pat_ID). Elapsed months since admission was calculated from the exact sampling dates. The Clinical Timepoint variable has been dummy coded with the baseline category (T0) implicit. Coefficients and associated statistics (Std.Err., z-value, p-value, and 95% confidence intervals) are shown for each predictor. Group Variance represents the variability attributed to differences between patients. Below are key model statistics:

• Observations: 100
• Groups (Patients): 52
• Min. group size: 1
• Max. group size: 3
• Mean group size: 1·9
• Scale: 0·1636
• Log-Likelihood: -103·8776
• Convergence: Yes

GFAP: Glial fibrillary acidic protein

Supplemental Figure 1: Study and Analysis Overview

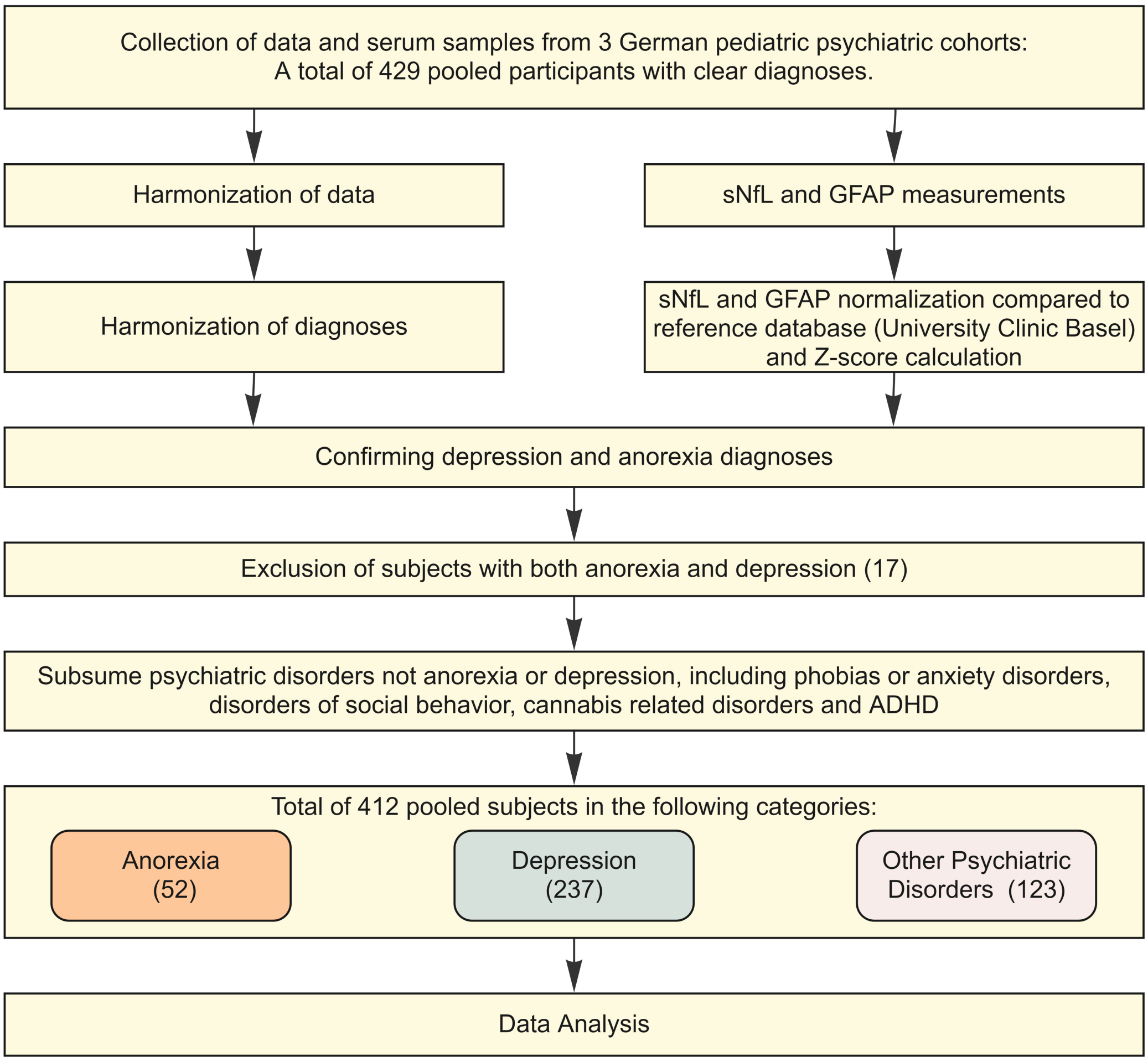

Supplemental Figure 1: Study and Analysis Overview.

This figure outlines the study design, including data collection from three German pediatric psychiatric cohorts, data harmonization, cohort definition, and exclusion criteria. The final analysis included 412 subjects across three groups: anorexia (52), depression (237), and other psychiatric disorders (123).

ADHD: Attention-Deficit/Hyperactivity Disorder; BMI: Body Mass Index, GFAP: Glial Fibrillary Acidic Protein, sNfL: Serum Neurofilament Light Chain.

Supplemental Figure 2: Elevated sNfL and GFAP Concentrations in Pediatric Anorexia Patients

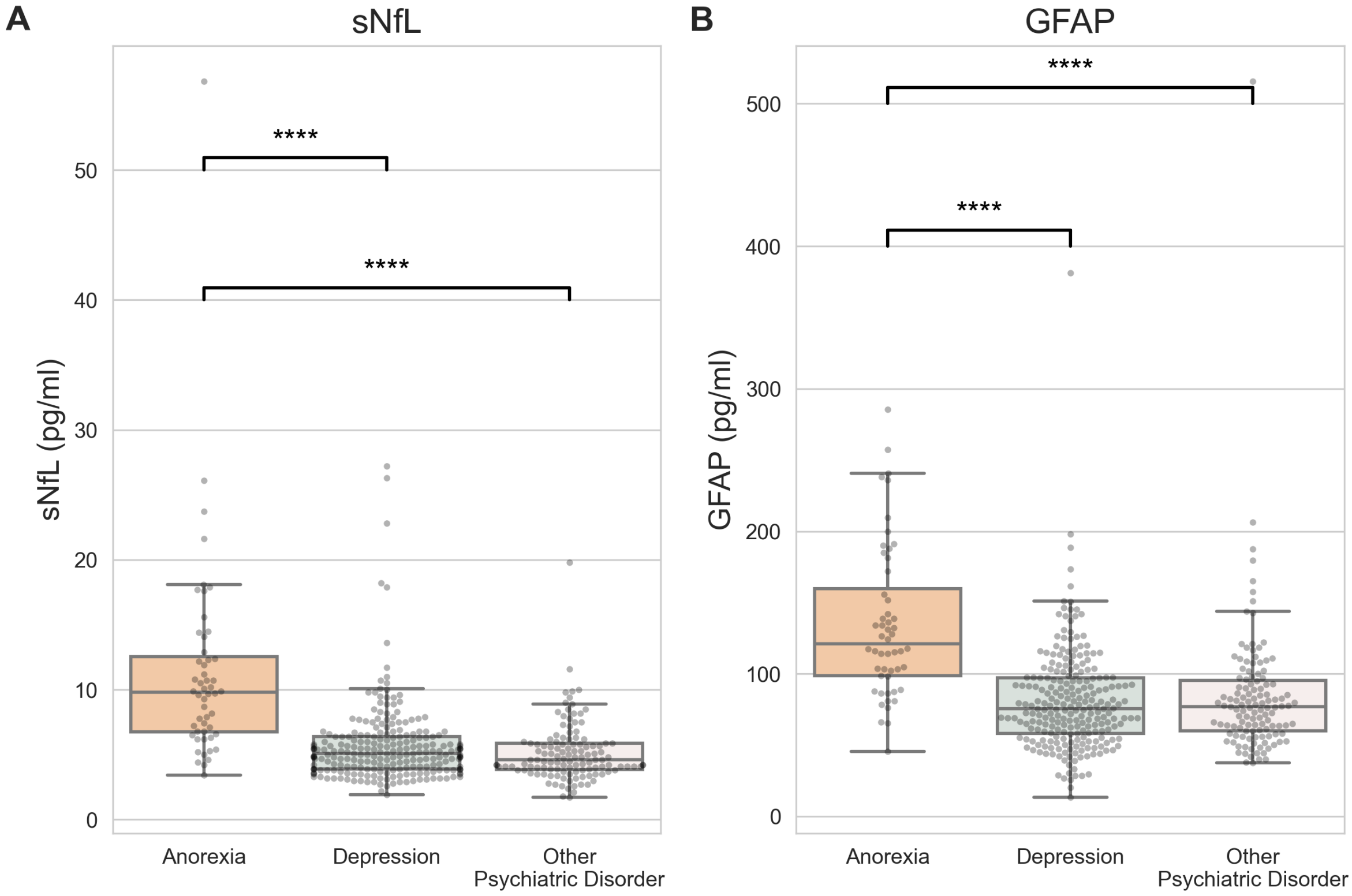

Supplemental Figure 2: Elevated sNfL and GFAP Concentrations in Pediatric Anorexia Patients.

(A) sNfL concentrations are shown using combined boxplots and swarm plots for patients with anorexia (orange), depression (green), and other psychiatric disorders (light pink). Anorexia patients exhibit significantly higher sNfL levels compared to the other groups.

(B) Similarly, GFAP concentrations are presented for the same patient groups, with anorexia patients again showing significantly higher values.

The boxplot center line represents the median, the box limits correspond to the upper and lower quartiles, and the whiskers indicate 1.5 times the interquartile range. Each grey point represents an individual patient's value.Post-hoc pairwise comparisons were calculated using Dunn's test with relevant differences marked with asterixes. Kruskal-Wallis test results: (A) sNfL, $H(2) = 70{\cdot}20$, $p < 0{\cdot}0001$; (B) GFAP, $H(2) = 58{\cdot}39$, $p < 0{\cdot}0001$

GFAP: Glial Fibrillary Acidic Protein, sNfL: Serum Neurofilament Light Chain

Supplemental Figure 3: sNfL and GFAP Concentrations for Anorexia Nervosa and Depression Patients by Weight Categories

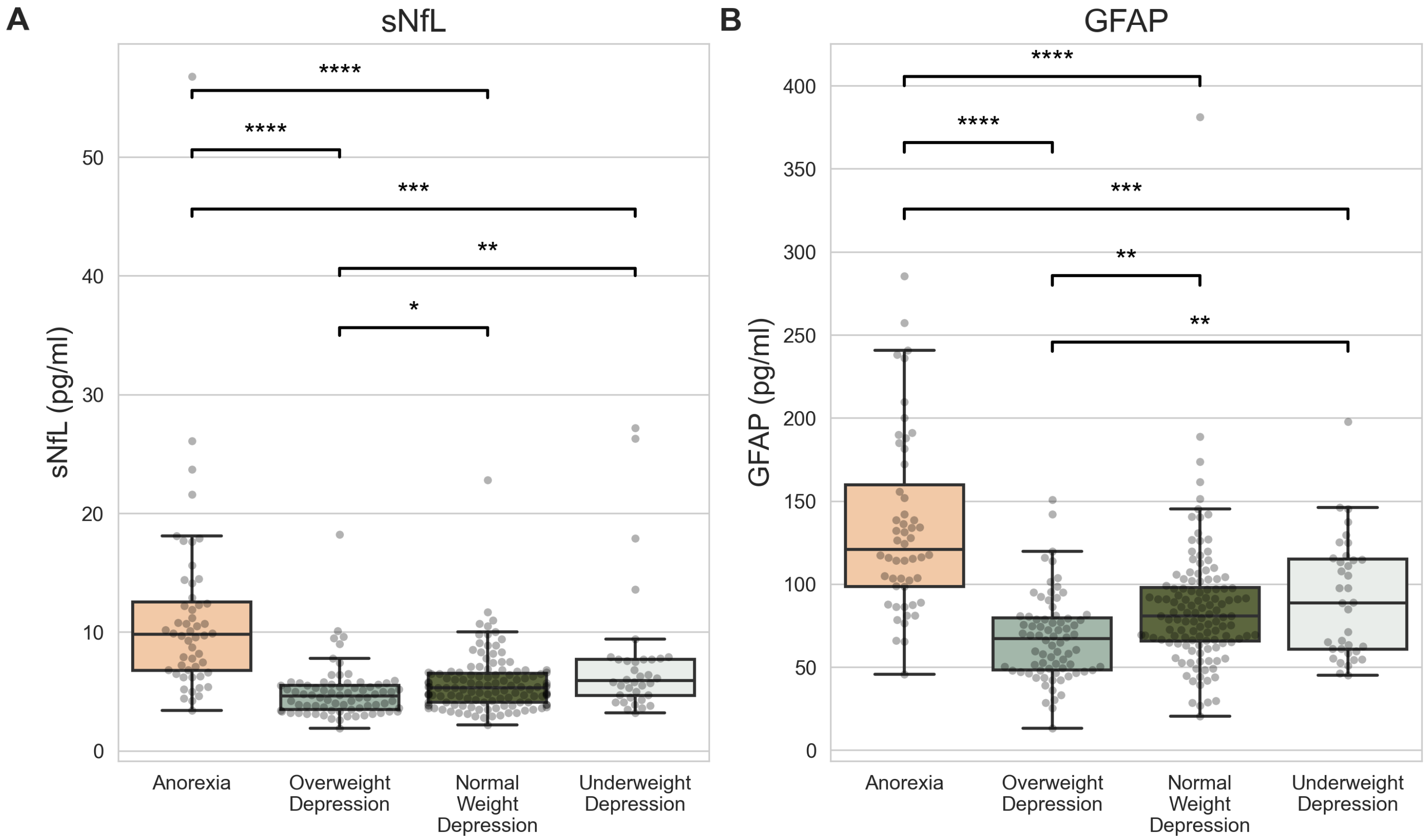

Supplemental Figure 3: sNfL and GFAP Concentrations for Anorexia Nervosa and Depression Patients by Weight Categories.

(A) The combined boxplot and swarmplot show sNfL concentrations (pg/mL) for anorexia nervosa patients (orange) and depression patients stratified by weight categories: overweight (turquoise), normal weight (green), and underweight (light gray). Anorexia patients show significantly higher sNfL concentrations compared to all weight-stratified depression groups. Additionally, overweight depression patients exhibit significantly lower sNfL concentrations than normal and underweight depression patients.

(B) GFAP concentrations (pg/mL) are presented for the same patient groups, with anorexia nervosa patients having significantly higher GFAP concentrations compared to all depression patient weight categories. Overweight depression patients show significantly lower GFAP concentrations than underweight depression patients.

The boxplot center line represents the median, the box limits correspond to the upper and lower quartiles, and the whiskers indicate 1.5 times the interquartile range. For AN versus underweight depression, Cohen's d is ≈0.53 for raw sNfL and ≈0.91 for raw GFAP. Each grey point represents an individual patient's value. Post-hoc pairwise comparisons were calculated using Dunn’s test. Significant results from Dunn’s test are indicated with asterisks above the relevant comparisons. Kruskal-Wallis test results: (A) sNfL, $H(3)=74{\cdot}17$, $p<0{\cdot}001$; (B) GFAP, $H(3)=71{\cdot}92$, $p<0{\cdot}001$.

GFAP: Glial Fibrillary Acidic Protein, sNfL: Serum Neurofilament Light Chain

Supplemental Figure 4: ROC Analysis of Serum Biomarkers Distinguishing Anorexia Nervosa from Underweight Depression

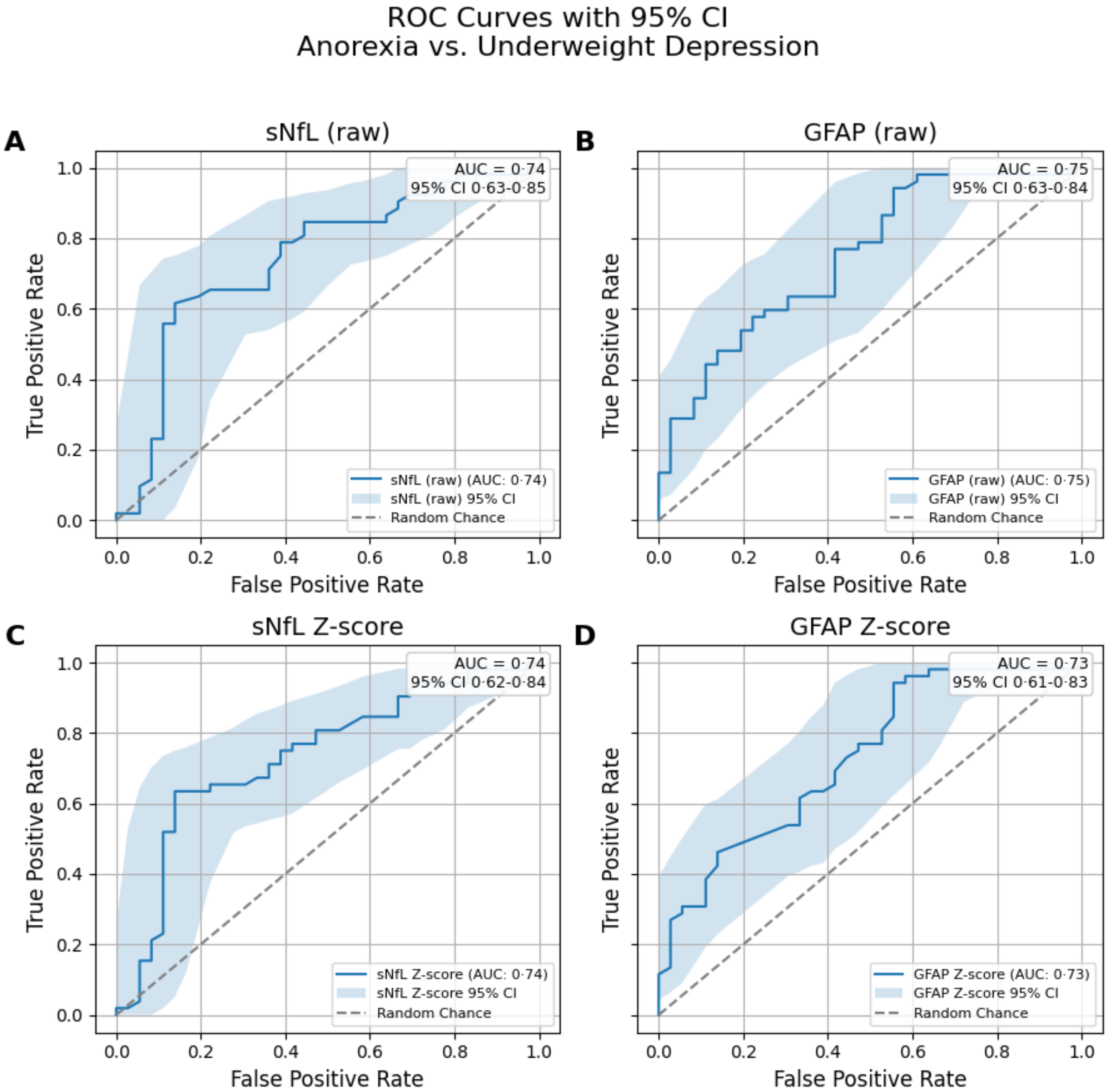

Supplemental Figure 4: ROC Analysis of Serum Biomarkers Distinguishing Anorexia Nervosa from Underweight Depression

For detailed legend see next page.

Supplemental Figure 4: ROC Analysis of Serum Biomarkers Distinguishing Anorexia Nervosa from Underweight Depression

Receiver Operating Characteristic (ROC) curves with 95% confidence intervals (CIs) for distinguishing anorexia nervosa (AN) from underweight depression based on sNfL and GFAP measurements. Shaded areas indicate the 95% CIs derived from 1000 bootstrap resamples. All reported thresholds and performance metrics (sensitivity, specificity) reflect the Youden's J criterion. (A) sNfL (Raw) shows an AUC of 0·74 (95% CI [0·622, 0·844]) with a mean threshold of 8·077 (95% CI [6·200, 9·700]), mean sensitivity of 0·671 (95% CI [0·500, 0·904]), mean specificity of 0·826 (95% CI [0·556, 0·972]), and mean Youden's J of 0·498 (95% CI [0·335, 0·647]).

(B) GFAP (Raw) yields an AUC of 0·75 (95% CI [0·648, 0·844]) with a mean threshold of 95·556 (95% CI [65·300, 132·100]), mean sensitivity of 0·797 (95% CI [0·461, 1·000]), mean specificity of 0·644 (95% CI [0·333, 0·944]), and mean Youden's J of 0·440 (95% CI [0·297, 0·592]).

(C) sNfL Z-score has an AUC of 0·74 (95% CI [0·615, 0·834]) with a mean threshold of 1·046 (95% CI [0·175, 1·440]), mean sensitivity of 0·649 (95% CI [0·500, 0·846]), mean specificity of 0·849 (95% CI [0·639, 0·972]), and mean Youden's J of 0·498 (95% CI [0·331, 0·656]).

(D) GFAP Z-score exhibits an AUC of 0·73 (95% CI [0·624, 0·831]) with a mean threshold of 0·654 (95% CI [-0·050, 1·774]), mean sensitivity of 0·841 (95% CI [0·423, 1·000]), mean specificity of 0·580 (95% CI [0·333, 0·944]), and mean Youden's J of 0·421 (95% CI [0·280, 0·571]).

For details see Supplemental Table 5.

AN = Anorexia Nervosa; AUC = Area Under the Curve; CI = Confidence Interval; GFAP = Glial Fibrillary Acidic Protein; ROC = Receiver Operating Characteristic; sNfL: Serum Neurofilament Light Chain; Youden's J = Youden's Index.

Supplemental Figure 5: Relationship between Premorbid BMI-SDS Differences and sNfL or GFAP Z-Scores in Anorexia Nervosa

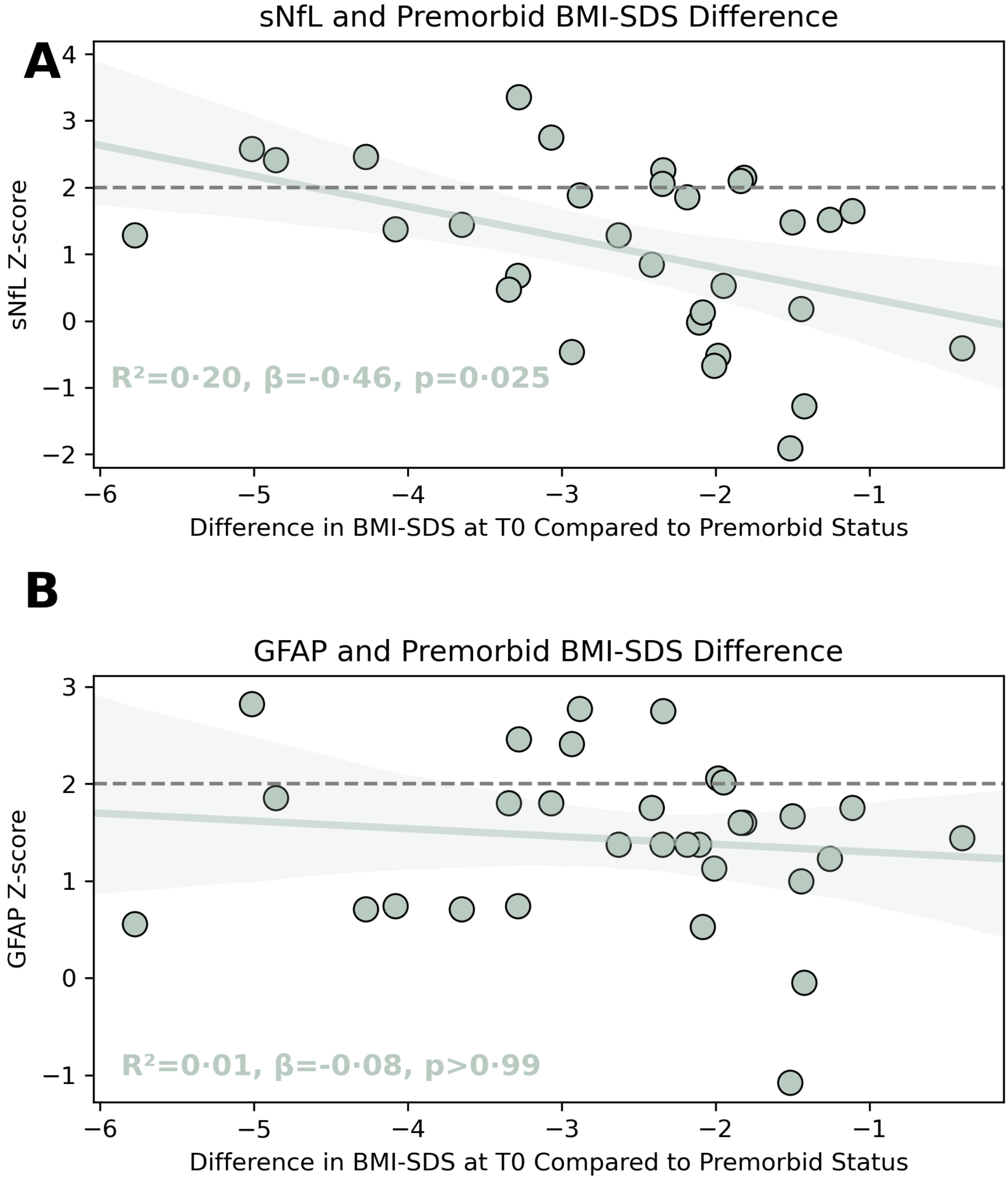

Supplemental Figure 5: Relationship between Premorbid BMI-SDS Differences and sNfL or GFAP Z-Scores in Anorexia Nervosa

A) The scatterplot displays the relationship between the difference in premorbid BMI-SDS and BMI-SDS at T0 (x-axis) and sNfL Z-scores at T0 (y-axis) in patients with Anorexia Nervosa (AN). The regression analysis indicates a significant negative correlation (β=-0·45, R^2=0·20, p=0·0248), suggesting that greater weight loss (larger negative difference in BMI-SDS) is associated with higher sNfL Z-scores.

(B) The second scatterplot shows the relationship between the same BMI-SDS difference and GFAP Z-scores at T0. No significant relationship was observed (β=-0·08, R^2=0·01, p>0·99).

The sNfL model R^2=0.20 corresponds to approximately 20% variance explained and a moderate effect size (Cohen's $f^2 \approx 0.25$; $|r| \approx 0.44$).

BMI-SDS: Body Mass Index Standard Deviation Score; GFAP: Glial Fibrillary Acidic Protein, sNfL: Serum Neurofilament Light Chain

Supplemental Figure 6: Correlation Matrix of sNfL and GFAP Across Clinical Stages in Anorexia Patients

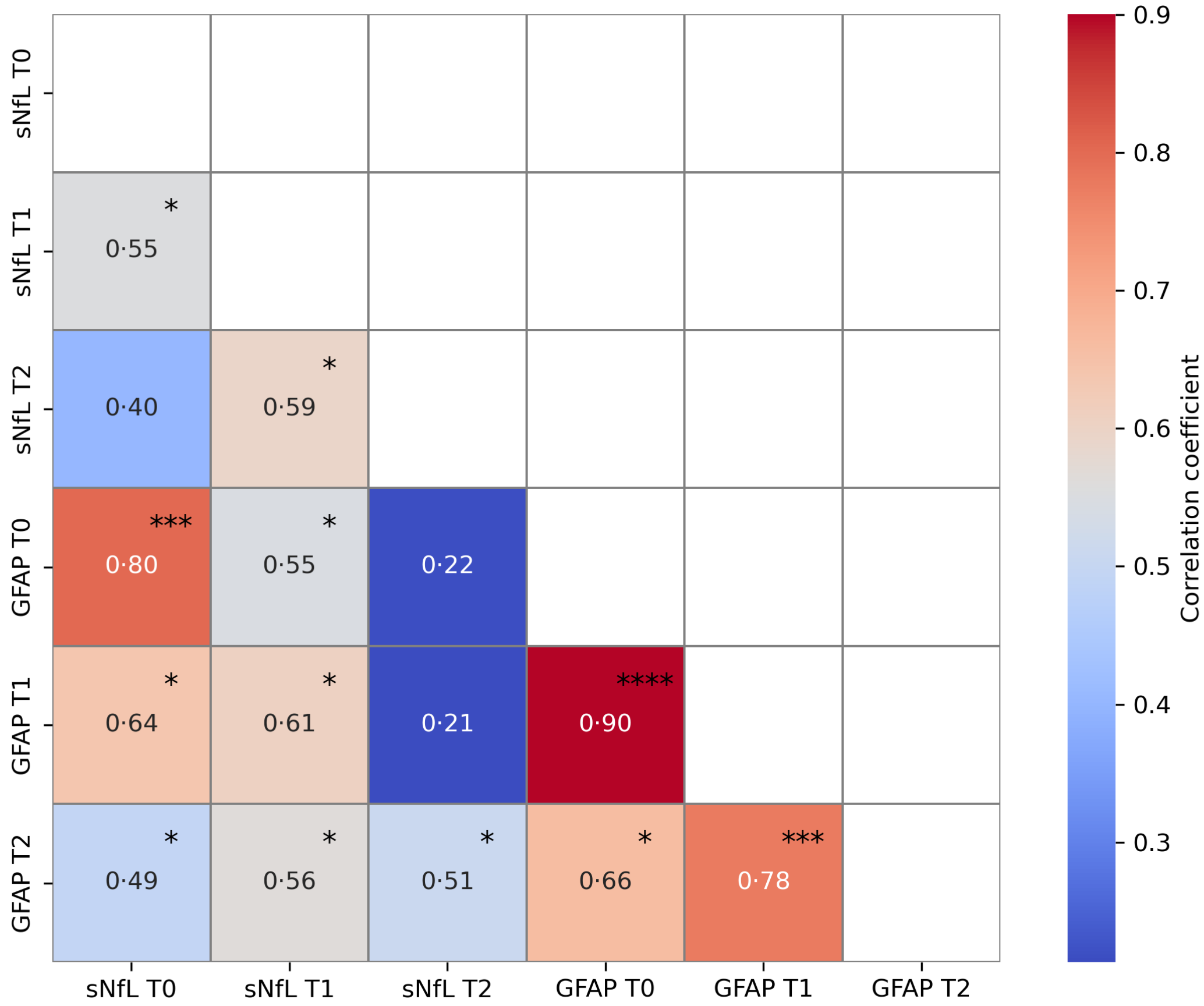

Supplemental Figure 6: Correlation Matrix of sNfL and GFAP Across Clinical Stages in Anorexia Patients

Correlation matrix showing Pearson correlation coefficients between sNfL and GFAP Z-scores at T0, T1, and T2 for anorexia patients with measurements at all timepoints (n=17). Timepoints are defined as T0 (admission for inpatient treatment), T1 (achievement of half of target weight), and T2 (after long-term weight regain and being close to target weight). The analysis was corrected for multiple comparisons using the false discovery rate method (Benjamini-Hochberg). High correlations were observed between sNfL and GFAP at T0, as well as within GFAP measurements across timepoints, with weaker correlations for sNfL across timepoints. Significant correlations are indicated by asterisks (*p<0·05, ***p<0·001).

GFAP: Glial Fibrillary Acidic Protein, sNfL: Serum Neurofilament Light Chain

Supplemental Figure 7: Development of sNfL and GFAP Concentrations by Clinical Stage in Pediatric Anorexia Patients

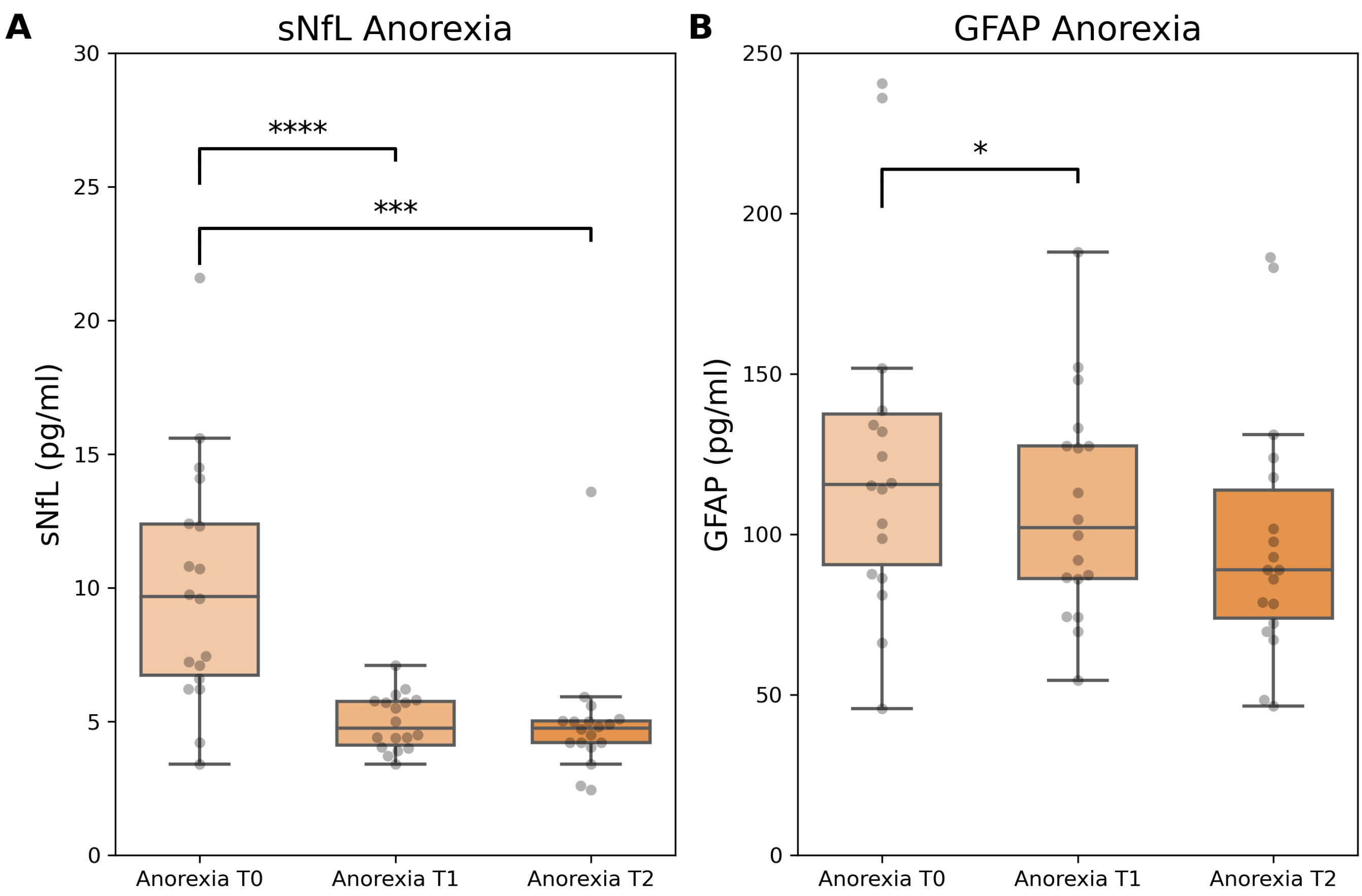

Supplemental Figure 7: Development of sNfL and GFAP Concentrations by Clinical Stage in Pediatric Anorexia Patients

(A) The combined boxplot and swarmplot show the development of sNfL concentrations (pg/mL) in pediatric anorexia patients during clinical recovery at T0 (admission for inpatient treatment), T1 (achievement of half of target weight), and T2 (after long-term weight regain and being close to target weight). sNfL concentrations are significantly higher at T0 compared to both T1 and T2.

(B) GFAP concentrations (pg/mL) are presented for the same patient groups. T0 GFAP concentrations are significantly higher than T1 concentrations, with no further significant differences.

The boxplot center line represents the median, the box limits correspond to the upper and lower quartiles, and the whiskers indicate 1.5 times the interquartile range. Each grey point represents an individual patient's value. Post-hoc pairwise comparisons were calculated using Wilcoxon signed-rank tests. Significant results from Wilcoxon tests are indicated with asterisks above the relevant comparisons.Friedman Test results: (A) sNfL, chi-square=22·33, p<0·001; (B) GFAP, chi-square=7·11, p=0·029.

GFAP: Glial Fibrillary Acidic Protein, sNfL: Serum Neurofilament Light Chain
